# A novel antisense lncRNA, LPCRL, functions as a molecular scaffold for the USP15/MIB1 complex to promote primary cisplatin resistance and tumor progression in lung squamous cell carcinoma

**DOI:** 10.1186/s13046-026-03721-7

**Published:** 2026-05-01

**Authors:** Peng Luo, Dapeng Lu, Shuang Zhang, Wenqian Dong, Kai Fang, Shihao Yu, Bing He, Maoxin Zhu, Yuee Wang, Xianliang Jiang, Baolong Wang

**Affiliations:** 1 Department of Laboratory Medicine, The First Affiliated Hospital of USTC, Division of Life Sciences and Medicine, University of Science and Technology of China, Hefei, 230001 China; 2 Core Unit of National Clinical Research Center for Laboratory Medicine, Hefei, 230001 China; 3 Department of Pathology, The First Affiliated Hospital of USTC, Division of Life Sciences and Medicine, University of Science and Technology of China, Hefei, 230001 China; 4 Department of Thoracic Surgery, The First Affiliated Hospital of USTC, Division of Life Sciences and Medicine, University of Science and Technology of ChinaThe First Affiliated Hospital of USTC, Division of Life Sciences and Medicine, University of Science and Technology of China, Hefei, 230001 China

**Keywords:** Lung squamous cell carcinoma, Cisplatin resistance, Antisense long noncoding RNA, LPCRL, USP15/MIB1 complex, Molecular scaffold

## Abstract

**Background:**

Platinum-based chemotherapy remains the first-line treatment for advanced lung squamous cell carcinoma (LUSC), but its efficacy is often hindered by the development of chemoresistance. Although long noncoding RNAs (lncRNAs) are recognized as regulators of tumor progression and drug resistance, the functional contribution of natural antisense transcripts (NATs), a major subclass of lncRNAs involved in cisplatin resistance in LUSC, remains poorly understood.

**Methods:**

Patient-derived xenograft (PDX) models of LUSC were established and treated with cisplatin to identify cisplatin-resistant and cisplatin-sensitive tumor tissues. LncRNA microarray profiling was used to identify transcripts associated with cisplatin resistance. The functional role of a candidate lncRNA, termed LPCRL (LUSC primary cisplatin resistance-associated LncRNA), was assessed in vitro via MTT, flow cytometry, colony formation, and Transwell migration assays. Its effects on tumor growth and metastasis were further validated in vivo. Mechanistic insights were gained through RNA pull-down, silver staining, RNA immunoprecipitation (RIP), coimmunoprecipitation (Co-IP), and Western blot analyses. Finally, the therapeutic potential of LPCRL-targeting siRNA was assessed in a LUSC PDX model.

**Results:**

We found that LPCRL was significantly upregulated in primary cisplatin-resistant PDX tissues. Functionally, LPCRL promoted primary cisplatin resistance and enhanced the proliferation and migration of LUSC cells both in vitro and in vivo. Mechanistically, LPCRL functions as a molecular scaffold to facilitate the interaction between MIB1 and USP15. This complex enables USP15 to deubiquitinate MIB1, thereby increasing MIB1 stability and promoting its nuclear export. The subsequent cytoplasmic accumulation of MIB1 enhances the ubiquitination of DLL4, leading to Notch pathway activation and upregulation of the downstream effector HES1. Importantly, intratumoral administration of LPCRL-targeting siRNA in PDX models suppressed tumor growth and sensitized tumors to cisplatin in vivo.

**Conclusions:**

Our study revealed that LPCRL promotes LUSC malignancy and cisplatin resistance via the USP15/MIB1/Notch axis, highlighting LPCRL as a promising therapeutic target.

**Graphical abstract:**

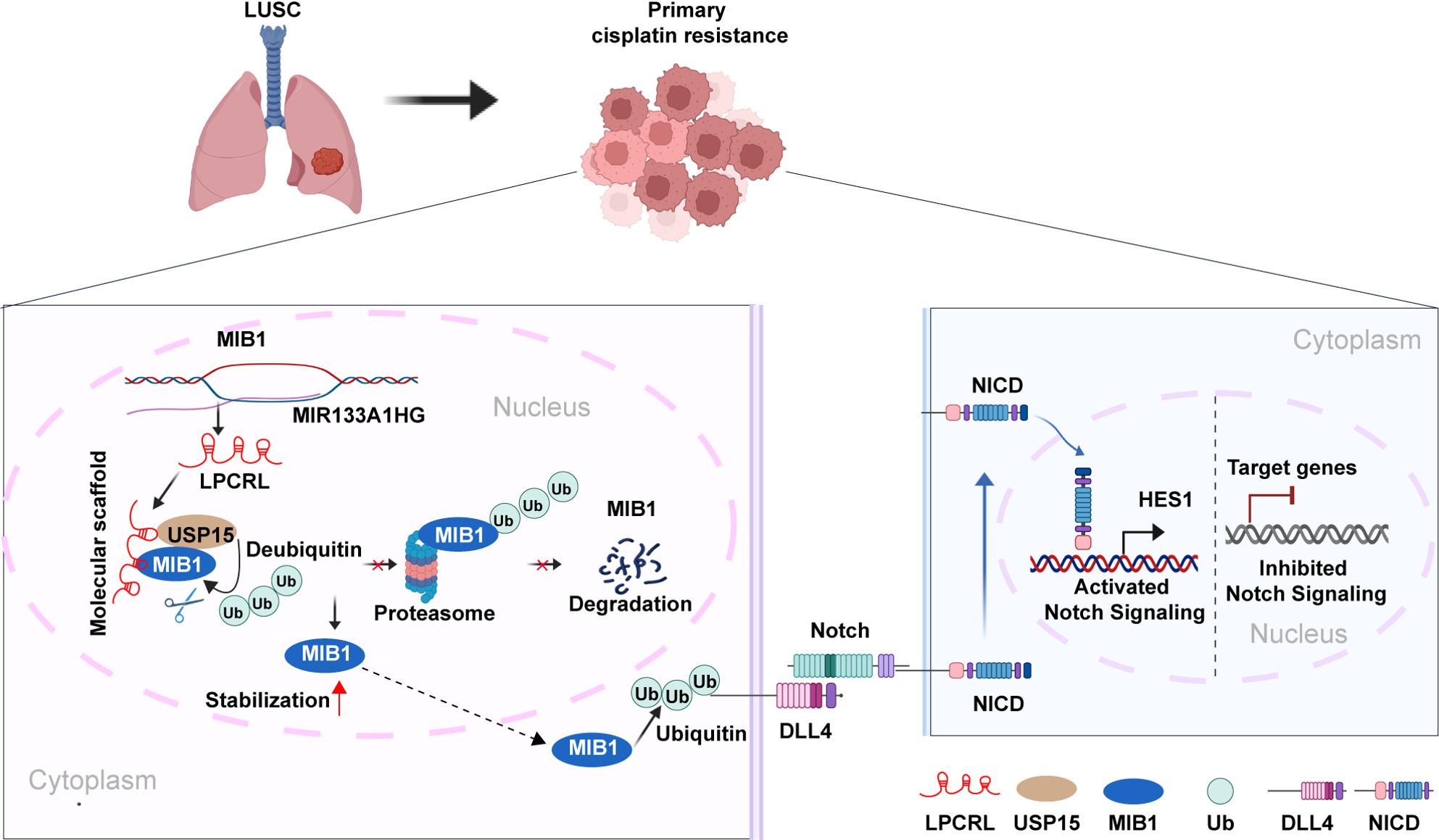

**Supplementary Information:**

The online version contains supplementary material available at 10.1186/s13046-026-03721-7.

## Introduction

Non-small cell lung cancer (NSCLC) accounts for 80%-85% of all lung cancer cases, with the squamous cell subtype comprising 20%-30% of NSCLCs [1]. Unfortunately, most LUSC patients are diagnosed at advanced stages, resulting in high mortality rates [2]. Although targeted therapies and immunotherapies have transformed the treatment landscape for LUSC [3], platinum-based chemotherapy remains the cornerstone of first-line treatment for advanced cases [4, 5]. However, its efficacy is often limited, with objective response rates of only 30%-40%, primarily due to intrinsic resistance [6, 7]. Therefore, elucidating the molecular mechanisms underlying primary platinum resistance in LUSC is critical for developing novel therapeutic strategies aimed at improving patient outcomes.

Long noncoding RNAs (LncRNAs) are defined as RNA transcripts longer than 200 nucleotides that lack protein-coding capacity but are critically involved in regulating diverse biological processes in lung cancer [8–11]. Antisense lncRNAs, a major subclass of lncRNAs, are transcribed from the complementary strand of protein-coding or noncoding genes. They account for approximately 50%-70% of all annotated lncRNAs and are widely distributed across both eukaryotic and prokaryotic genomes. Notably, antisense lncRNAs can regulate gene expression at multiple levels, including the pretranscriptional, transcriptional, and posttranscriptional levels, through interactions with DNA, RNA, or proteins [12], and emerging evidence has linked these transcripts to various aspects of cancer biology, including drug resistance [10], metabolic reprogramming [11], phase separation [13], cell proliferation, and metastasis [14]. However, despite accumulating evidence implicating various lncRNAs in cisplatin resistance [15–17], including in lung adenocarcinoma [18], the functional roles and underlying mechanisms of antisense lncRNAs in primary cisplatin resistance specifically in LUSC remain largely unexplored. Patient-derived xenograft (PDX) models are widely recognized as robust preclinical tools that recapitulate intratumoral heterogeneity, preserve native tumor architecture, and reliably reflect drug responses and resistance mechanisms [19]. After serial passaging to the third generation (P3), these models reach a biological “plateau” with stabilized characteristics, enhancing their reproducibility for drug studies [20].

In this study, microarray analysis of the lncRNA expression profiles of third-generation xenograft tumors revealed a clear transcriptional distinction between the cisplatin-sensitive and cisplatin-resistant groups via principal component analysis. We identified a markedly upregulated antisense transcript, uc002ktr.3, hereafter referred to as LPCRL (LUSC primary cisplatin resistance-associated LncRNA), in the cisplatin-resistant cohort. This transcript is derived from the *MIR133A1HG* gene locus, which overlaps the antisense strand of the twelfth intron of *MIB1*. Functional studies demonstrated that LPCRL enhance cisplatin resistance, promote proliferation, and facilitate metastasis in LUSC cells. Mechanistically, LPCRL functions as a molecular scaffold that directly mediates the interaction between MIB1 and USP15. This promotes USP15-mediated deubiquitination of nuclear MIB1. This posttranslational modification stabilizes MIB1 and promotes its nuclear export. The resulting cytoplasmic accumulation of MIB1 activates the Notch signaling pathway. Importantly, the siRNA-mediated silencing of LPCRL in vivo significantly suppressed tumor growth and migration while simultaneously enhancing cisplatin sensitivity. In summary, our study reveals a pivotal role for the LPCRL/USP15/MIB1/Notch signaling axis in promoting cisplatin resistance and tumor progression in LUSC, identifying LPCRL as a promising molecular target for therapeutic intervention.

## Materials and methods

### Clinical specimens

Patient-derived xenograft (PDX) models were established using tumor tissues from patients diagnosed with LUSC at The First Affiliated Hospital of the University of Science and Technology of China. All tumor samples were collected with approval from the Ethics Committee of the University of Science and Technology of China (Approval No. 2019-N(H)-128), and written informed consent was obtained from all participants.

### Establishment of PDX models and cisplatin chemosensitivity testing

All animal procedures were conducted in accordance with the Declaration of Helsinki and were approved by the Institutional Animal Care and Use Committee of the University of Science and Technology of China (Approval No. 2019-N (A)-179). PDX models were generated as previously described [21, 22]. Briefly, fresh tumor tissues were sectioned into ~ 3 mm³ fragments and implanted subcutaneously into the flanks of BALB/c nude mice. Following the same method, the transplanted tumors were serially passaged to the third generation. Once the third-generation xenograft tumor volume reached 50–200 mm³, 3–5 mice per model were randomized into treatment or control groups. The tumor volume was calculated as (length × width^2)/2.

Cisplatin (20 mg/mL; Jiangsu Hansoh Pharmaceutical Co., Ltd.) was diluted to 0.5 mg/mL in saline and administered intraperitoneally at 5 mg/kg once weekly for 3 consecutive weeks (treatment group). The control mice received an equivalent volume of PBS. The injection volume was standardized at 0.2 mL per 20 g body weight. A tumor growth inhibition rate (TIR = 1-(average tumor volume in the cisplatin group/average tumor volume in the PBS group)×100%) ≥ 50% was considered cisplatin sensitive; <50% was considered resistant.

### Establishment of primary LUSC cells

Primary LUSC cells were established as follows. Briefly, fresh LUSC tumor tissue from surgical resection was placed in cold preservation medium (DMEM with antibiotics/antimycotics) on ice. The tissue was minced into 1–2 mm³ fragments and digested in a collagenase solution (200 U/mL, Sigma Aldrich, Saint Louis, MO, USA) at 37 °C with periodic shaking or resuspension. The resulting cell suspension was filtered through a 70 μm cell strainer, and the filtrate was centrifuged. The cell pellet was washed with PBS, resuspended in culture medium (RPMI 1640 medium (HyClone) with 10% FBS), and seeded. Cultures were maintained in a humidified incubator at 37 °C with 5% CO₂, with regular medium changes to remove nonadherent cells and debris. Once the cells reached 80%-90% confluence, they were passaged with trypsin-EDTA (Beyotime).

### LncRNA microarray and bioinformatic analysis

Total RNA was extracted from cisplatin-resistant and cisplatin-sensitive PDX samples for microarray analysis via the Arraystar Human LncRNA Microarray V3.0 (Agilent Technologies). Feature extraction was performed with Agilent Feature Extraction software (v11.0.1.1), and quantile normalization and data processing were conducted via GeneSpring GX v12.1 (Agilent Technologies). Differentially expressed lncRNAs were identified by a fold change > 1.5 and a *p* value < 0.05. Principal component analysis and volcano plots were generated via in-house scripts.

### Cell culture

SK-MES-1 and HEK293T cell lines were obtained from the Cell Bank of the Chinese Academy of Sciences (Shanghai, China). The NCI-H520 cell line was purchased from the American Type Culture Collection (ATCC; Manassas, VA, USA). LUSC primary cells were established in our laboratory. NCI-H520 and primary cells were cultured in RPMI-1640 medium (HyClone) supplemented with 10% fetal bovine serum (FBS; Proteintech). SK-MES-1 and HEK293T cells were cultured in DMEM/high-glucose medium (HyClone) supplemented with 10% FBS. All the cells were maintained at 37 °C in a humidified incubator with 5% CO₂ and passaged using 0.25% trypsin-EDTA (Beyotime).

### RNA extraction, reverse transcription and quantitative PCR (RT‒qPCR)

Total RNA was extracted via TRIzol Reagent (Life Technologies, CA, USA). Nuclear and cytoplasmic RNA fractions were isolated via nuclear and cytoplasmic extraction reagents (Invitrogen, NY, USA). Complementary DNA (cDNA) was synthesized with the PrimeScript™ RT‒PCR Kit (Takara Bio, Otsu, Shiga, Japan), and quantitative PCR was performed via SYBR^®^ Select Master Mix (Vazyme, Nanjing, China) on an ABI 7500 real-time PCR system (Thermo Fisher Scientific, USA). The primer sequences are listed in Supplementary Table S1.

### Small interfering RNA (siRNA), antisense oligonucleotides, plasmid construction and cell transfection

Small interfering RNAs (siRNAs) were obtained from Generalbiol (Shanghai, China). Antisense oligonucleotides (ASOs) were obtained from RiboBio (Guangzhou, China). Plasmid vectors (pCMV-LPCRL, pCMV-LPCRL-M1, pCMV-LPCRL-M2, pCMV-LPCRL-M3, pCMV-HA-USP15, pCMV-Myc-MIB1, pCMV-Myc-MIB1-Del MZM/REP/ANK/RNG, and empty pCMV vector) were obtained from the Miaoling Plasmid Platform (Wuhan, China). siRNAs and ASOs were transfected via a Lipofectamine™ 2000 kit (Invitrogen, Carlsbad, CA, USA) in accordance with the manufacturer’s protocol. Plasmid transfection was performed using a PEI MW40000 (Yeasen Biotechnology, Shanghai, China). All the siRNA sequences used are listed in Supplementary Table S2.

### Lentiviral transfection

Lentiviral-shLPCRL and Lentiviral-LPCRL were obtained from Hanheng Biological Company (Shanghai, China). Lentiviral (LV)-short hairpin negative control (shNC) and LV-sh LPCRL (sh LPCRL-1, sh LPCRL-2) were used to transfect SK-MES-1 cells. The short hairpin RNA sequences used to silence the LPCRL are listed in Supplementary Table S1. Lentiviral (LV)-LPCRL (oeLPCRL) was used to transfect NCI-H520 cells. Inducible cell sublines were established via puromycin selection and validated via RT‒qPCR.

### MTT and colony formation assays

For the MTT assays, 96-well plates were seeded with cells and incubated with MTT (5 mg/mL; Biosharp, Anhui, China) for 4 h. The absorbance was measured at 490 nm. For the colony formation assays, 1,000 cells/well were seeded in 6-well plates and cultured for 1–2 weeks. Colonies were fixed in 4% paraformaldehyde, stained with crystal violet (Sangon Biotech, Shanghai, China), and counted manually.

### Transwell migration assay

For the migration assays, 8 × 10⁴ cells in 200 µL of serum-free DMEM were seeded into the upper chambers of Transwell plates (8-µm pores; Corning, NY, USA). The lower chambers contained 800 µL of medium supplemented with 10% FBS as a chemoattractant. After 24–48 h, the migrated cells were fixed, stained with crystal violet, and quantified under a microscope.

### IC50 assay

The cells (8 × 10⁴ cells/well) were seeded in 96-well plates, allowed to adhere, and then treated with various concentrations of cisplatin for 48 h. IC₅₀ values were calculated via GraphPad Prism v9.5.0 on the basis of the logarithmic relationship between drug concentration and the cellular response.

### Flow cytometry apoptosis assay

Apoptosis was assessed via an Annexin V-FITC/PI Apoptosis Detection Kit (Keygen Biotech, Nanjing, China). After 24 h of cisplatin or paclitaxel treatment, the cells were washed with PBS, resuspended in 500 µL of binding buffer, and stained with 5 µL of Annexin V-FITC and 5 µL of propidium iodide (PI) for 10 min in the dark. Apoptosis was analyzed via a CytoFLEX flow cytometer and CytExpert software (Beckman Coulter). The cells were categorized as viable (FITC⁻/PI⁻), early apoptotic (FITC⁺/PI⁻), or late apoptotic/dead (FITC⁺/PI⁺).

### Fluorescence in situ hybridization (FISH)

Biotin-labeled LPCRL probes (Ribobio, Guangzhou, China) were used to detect LPCRL localization with a FISH detection kit (Ribobio). Fixed cells were hybridized with probes overnight, washed, stained with DAPI, and visualized via fluorescence microscopy. The tissue samples were cryosectioned at 4 μm after fixation in 4% paraformaldehyde for 1 h and processed similarly.

### RNA pull-down and RNA-binding protein immunoprecipitation (RIP) assays

The RNA pull-down assays used biotin-labeled LPCRL probes (RiboBio) and their antisense controls. SK-MES-1 cells were lysed in ice-cold lysis buffer (50 mM Tris-HCl, pH 8.0; 150 mM NaCl; 5 mM EDTA; 1% NP-40; 0.1% SDS; 1 mM DTT; 1× protease inhibitor cocktail; 0.1 U/µL RNase inhibitor) for 15 min on ice. The lysates were centrifuged at 12,000×g for 15 min, and the supernatants were incubated with 100 pmol of biotinylated oligonucleotides or 2 µg of antibodies (MIB1, USP15 for RIP) overnight at 4 °C. M-280 streptavidin Dynabeads (Invitrogen, 11206D, for RNA pull-down) or Protein G Dynabeads (Invitrogen, 10004D, for RIP) preblocked with 500 ng/µL yeast total RNA and 5% BSA were added for 2 h at room temperature. The beads were washed with lysis buffer and high-salt lysis buffer (500 mM NaCl). The purified RNAs were analyzed via RT‒qPCR, and the proteins were analyzed via western blotting after silver staining.

### 5′ and 3′ rapid amplification of cDNA ends (RACE)

RACE was performed via the SMARTer RACE 5′/3′ Kit (Takara). Total RNA from SK-MES-1 cells was used to synthesize 5′- and 3′-RACE-ready cDNA, which was amplified via nested PCR with universal primers and gene-specific primers (Supplementary Table S2).

### Western blot

Total protein was extracted via a Total Protein Extraction Kit (Bestbio, Shanghai, China) with protease and phosphatase inhibitors (Epizyme, Shanghai, China). Proteins were quantified and separated by SDS‒PAGE, transferred to PVDF membranes (Merck Millipore), blocked with 5% skim milk, and incubated with primary antibodies overnight, followed by incubation with secondary antibodies (Proteintech). Bands were detected via a Tanon multigel imaging system. The following antibodies were used: MIB1 (sc-393551, Santa Cruz, 1:500), USP15 (66310s, CST, 1:1000), c-PARP (ab32561, Abcam, 1:1000), PARP1 (13371-1-AP, Proteintech, 1:1000), γ-H2AX (CY6572, Abways, 1:1000), β-Actin (66009-1-Ig, Proteintech, 1:10000), GAPDH (60004-1-Ig, Proteintech, 1:10000), Ub (#20326, CST, 1:1000), Myc (60003-2-Ig, Proteintech, 1:2000), HA (51064-2-AP, Proteintech, 1:2000), DLL4 (21584-1-AP, Proteintech, 1:2000), LaminB1 (12987-1-AP, Proteintech, 1:2000), NICD (10062-2-AP, Proteintech, 1:2000), N-cadherin (22018-1-AP, Proteintech, 1:2000), PCNA (60097-1-Ig, Proteintech, 1:5000), Vimentin (10366-1-AP, Proteintech, 1:5000), and c-Myc (10828-1-AP, Proteintech, 1:5000).

### Immunoprecipitation (IP)

The cells were lysed in IP buffer supplemented with protease and phosphatase inhibitors, and the lysates were subsequently centrifuged. The supernatants were immunoprecipitated with antibodies against MIB1 (Santa Cruz), USP15 (CST), DLL4 (Proteintech), Myc (Proteintech), or HA (Proteintech) overnight at 4 °C. Immune complexes were captured with protein A/G magnetic beads (Biolinkedin, Shanghai, China), washed with ice-cold PBS, 20 µL of 1× loading buffer was added, the mixture was heated at 95 °C for 10 min, and the proteins were analyzed by western blotting.

### Animal experiments

Female NOD-SCID mice (4–6 weeks old; *n* = 6, 3 per group) and BALB/c nude mice (4–6 weeks old; *n* = 18, 9 per group) were obtained from Charles River Laboratories (Zhejiang, China). SK-MES-1 cells (5 × 10⁶ cells/mouse) transfected with si-LPCRL or si-NC were injected subcutaneously into BALB/c nude mice. Tumor growth was monitored every 5 days. After 4 weeks, the mice were euthanized, and the tumors were fixed in 10% formalin for pathological analysis.

For the metastasis assays, luciferase-labeled SK-MES-1 cells (1 × 10⁶) transfected with si-LPCRL or si-NC were injected via the tail vein into NOD-SCID mice. Lung metastases were monitored by bioluminescence imaging (IVIS Spectrum). After 3 weeks, the mice were euthanized, and the lungs were fixed and stained with H&E for histological confirmation.

### In vivo PDX model-based therapeutic study

When the PDX tumor volume reached 50–100 mm³, the mice were randomized into 5 groups (*n* = 8/group): (a) blank control (5% glucose); (b) si-NC; (c) si-LPCRL; (d) si-NC+cisplatin; and (e) si-LPCRL+cisplatin. Si-LPCRL or si-NC (10 µg/tumor/dose) in 5% glucose was administered intratumorally via in vivo jetPEI^®^ (Polyplus, France) every 5 days for 4 doses. Cisplatin (2.5 mg/kg) was administered intraperitoneally every 5 days for 3 doses in the combination groups. The mice were euthanized, and the tumors were excised, weighed, measured, and paraffin-embedded for IHC.

### Immunohistochemistry (IHC)

The tumor sections were deparaffinized, rehydrated, and subjected to antigen retrieval with sodium citrate (Beyotime). After blocking, the sections were incubated with primary antibodies (MIB1, Proteintech, 1:200; HES1, Bioss, Beijing, 1:200; γ-H2AX, Bioss, 1:200; Ki-67, Abcam, 1:200) overnight at 4 °C and then with HRP-labeled secondary antibodies (Boster). Staining was performed with DAB (Beyotime), and the sections were counterstained with hematoxylin. Images were captured with a Nikon microscope. Expression was quantified by the H score (staining intensity×percentage of positive cells). Intensities: 0 (none), 1 (light brown), 2 (brown), and 3 (dark brown). Positive cells: 0 (< 5%), 1 (5–25%), 2 (26–50%), 3 (51–75%), and 4 (76–100%).

### Statistical analysis

All the experiments were performed in triplicate. The data are presented as the means ± standard deviations (SDs). Comparisons between two groups were performed via two-tailed Student’s t tests. Paired samples were analyzed with paired t tests. A *p* value of less than 0.05 was considered statistically significant. Statistical analyses were conducted via SPSS (version 17.0).

## Results

### LPCRL is upregulated in primary cisplatin-resistant LUSC tissues

**Fig. 1:**
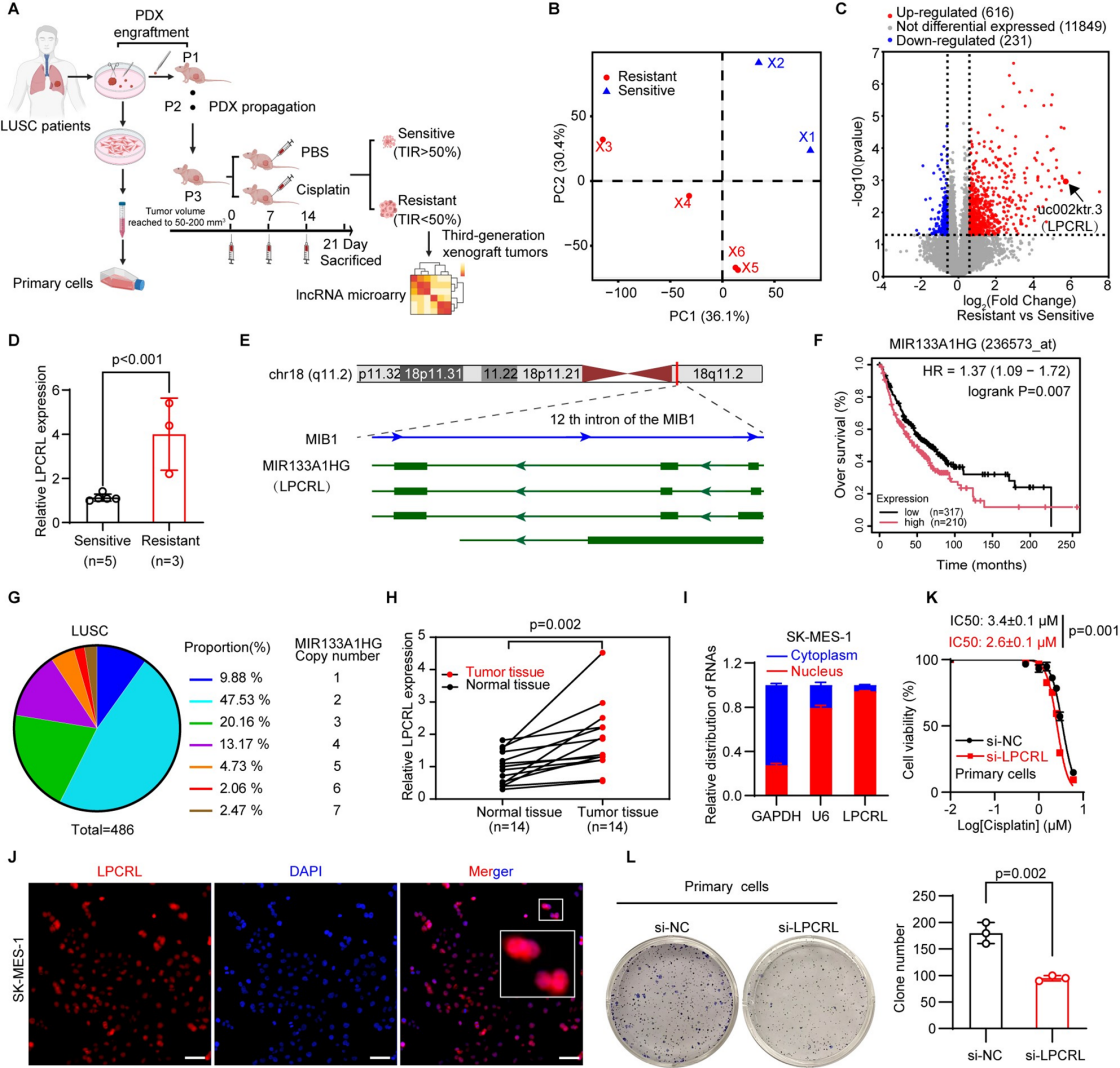
LPCRL is upregulated in primary cisplatin-resistant LUSC tissues. **A** Schematic illustration showing cisplatin or PBS treatment in patient-derived xenografts (PDXs) of lung squamous cell carcinomas (LUSC) and the establishment of primary cells. **B** Principal component analysis of long noncoding RNA (lncRNA) data from third-generation xenograft tumors. Each dot represents a sample, colored according to its response to cisplatin (blue, sensitive; red, resistant). **C** Volcano plots showing differentially expressed lncRNAs in the cisplatin-resistant group versus the cisplatin-sensitive group (fold change > 1.5; adjusted *p* value < 0.05). **D** RT‒qPCR analysis showing relative LPCRL expression in the cisplatin-resistant group (*n* = 3) and the cisplatin-sensitive group (*n* = 5). **E** Schematic of the genomic localization of MIR133A1HG (chr18q11.2), an antisense lncRNA hereafter referred to as LPCRL that overlaps the antisense strand of intron 12 of MIB1, on the basis of the UCSC Genome Browser. **F** Kaplan‒Meier analysis showing the association between MIR133A1HG expression and overall survival (OS). **G** Pie chart showing the absolute MIR133A1HG copy number distribution in LUSC (TCGA dataset, *n* = 486). **H** RT‒qPCR analysis showing relative LPCRL expression in LUSC tumor tissues and adjacent normal tissues (*n* = 14 pairs) (paired t test). **I** Subcellular localization of LPCRL in SK-MES-1 cells on the basis of nuclear and cytoplasmic RNA separation followed by RT‒qPCR analysis. GAPDH (cytoplasmic) and U6 (nuclear) were used as controls. **J** Representative fluorescence in situ hybridization (FISH) images showing LPCRL signals (red) in SK-MES-1 cells. Nuclei are stained with DAPI (blue). Scale bar: 50 μm. **K** The viability of primary cells transfected with si-LPCRL or si-NC was evaluated via the MTT assay after treatment with various concentrations of cisplatin for 48 h. **L** Colony formation assays showing the proliferative capacity of primary cells transfected with si-LPCRL or si-NC. Representative images (left panel) and quantitative analysis of colony numbers (right panel) are presented

To identify key lncRNAs associated with primary cisplatin resistance in LUSC, we established six LUSC PDX models, which were serially passaged to the third generation. After three cycles of cisplatin treatment, the models were classified into cisplatin-sensitive (tumor inhibition rate, TIR > 50%; *n* = 2) and cisplatin-resistant (TIR < 50%; *n* = 4) groups (Fig. 1A; Supplementary Fig. S1A, S1B). Microarray analysis of the lncRNA expression profiles of third-generation xenograft tumors revealed a clear transcriptional distinction between the cisplatin-sensitive and cisplatin-resistant groups via principal component analysis (Fig. 1B). Volcano plot analysis identified 847 differentially expressed lncRNAs (|fold change|>1.5, *p* < 0.05). Among these, uc002ktr.3, which we designated LPCRL (lung squamous cell carcinoma primary cisplatin resistance-associated long noncoding RNA), was the most significantly upregulated antisense lncRNA in cisplatin-resistant tissues (Fig. 1C). RT‒qPCR analysis confirmed significantly increased LPCRL expression in cisplatin-resistant tissues (*n* = 3) from eight additional PDX models, which were established and classified on the basis of cisplatin screening (Fig. 1D).

Bioinformatic analysis revealed that the LPCRL is located on chromosome 18 and is transcribed from the *MIR133A1HG* gene, which overlaps with the antisense strand of the twelfth intron of *MIB1* (Fig. 1E). Kaplan‒Meier analysis revealed that high MIR133A1HG expression was correlated with poorer overall survival in LUSC patients [23] (Fig. 1F). Analysis of the GDC TCGA LUSC cohort revealed that 42.6% (207/486) of patients harbored MIR133A1HG copy number gains (Fig. 1G). Given the role of copy number variations (CNVs) in driving gene expression and tumorigenesis [24, 25], we hypothesized that LPCRL expression would be elevated in LUSC. Consistent with these findings, RT‒qPCR confirmed significantly higher LPCRL levels in LUSC tissues than in paired normal adjacent tissues (Fig. 1H). This tumor-specific overexpression was further corroborated by fluorescence in situ hybridization (FISH), which revealed markedly stronger LPCRL signals in tumor cells than in stromal cells within the same tissue (Supplementary Fig. S1C). In summary, these data establish LPCRL as an antisense lncRNA that not only is highly expressed in LUSC but is also further upregulated in a cisplatin-resistant context, positioning it as a key mediator of LUSC resistance to cisplatin.

To molecularly characterize LPCRL, we performed 5’ and 3’ RACE in LUSC cells. Using a 5,912-nt reference sequence from the UCSC Genome Browser (which annotates four MIR133A1HG transcripts; Supplementary Fig. S1D), we identified a single 688-nt, single-exon transcript in SK-MES-1 cells. This variant was confirmed by ORF Finder to lack protein-coding potential (Supplementary Fig. S1E) and constitutes a partial segment of the MIR133A1HG-204 isoform (Supplementary Fig. S1F). RNA FISH and subcellular fractionation assays revealed that LPCRL is predominantly localized in the nucleus of both SK-MES-1 and NCI-H520 cells (Fig. 1I and J; Supplementary Fig. S1G, S1H), two widely used cell lines in LUSC research. Given the high expression of LPCRL in LUSC, we performed siRNA-mediated knockdown in primary LUSC cells established in our laboratory (Supplementary Fig. S1I). LPCRL knockdown significantly increased cisplatin sensitivity and reduced cell proliferation (Fig. 1K and L). Taken together, our results define LPCRL as a nuclear-enriched antisense lncRNA associated with cisplatin resistance and cell proliferation in LUSC.

### LPCRL promotes cisplatin resistance, proliferation and migration in LUSC

**Fig. 2:**
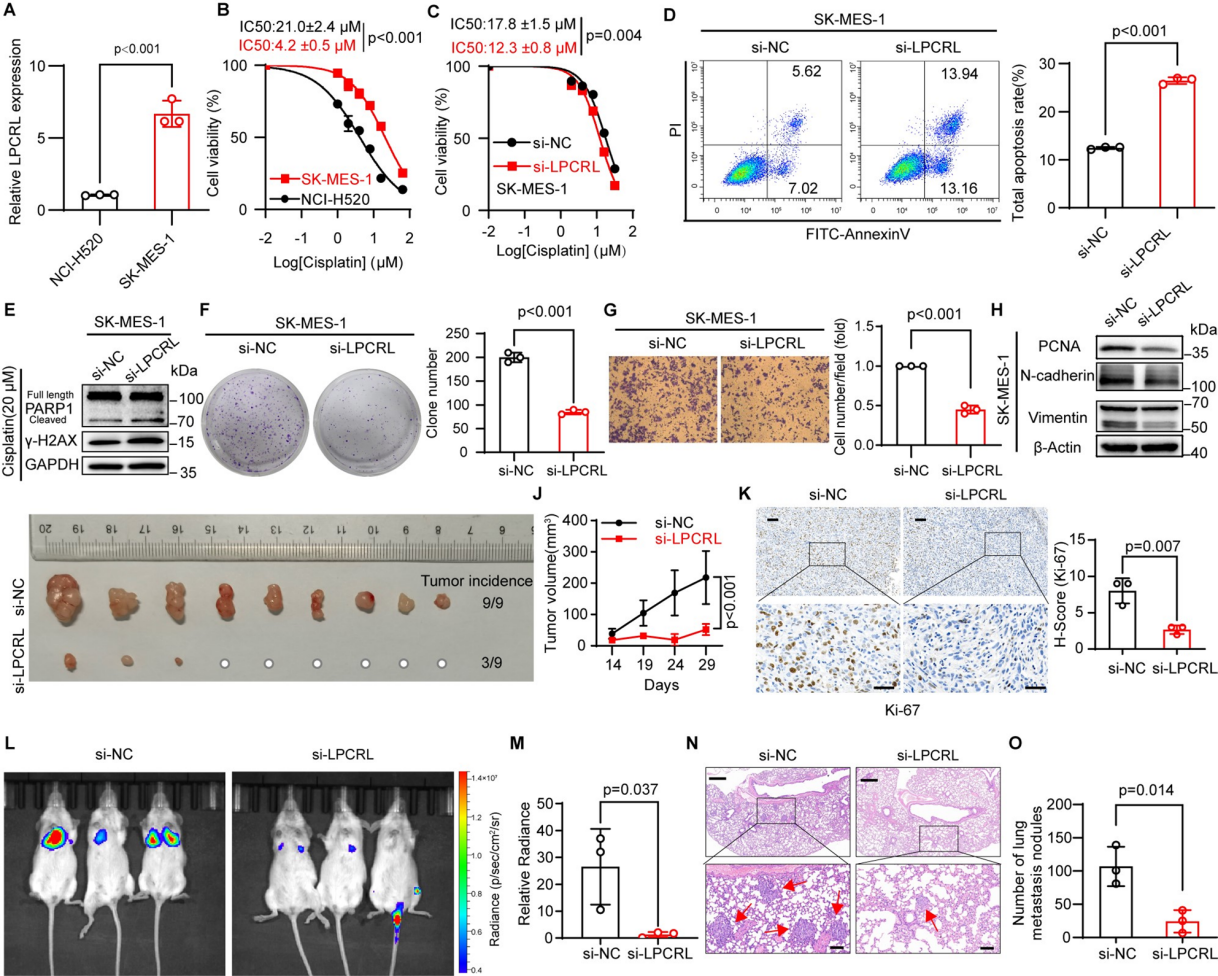
LPCRL promotes cisplatin resistance, cell proliferation and migration in SK-MES-1 cells. **A** RT‒qPCR analysis of endogenous LPCRL expression in SK-MES-1 and NCI-H520 cells. **B-C** Cell viability was evaluated by the MTT assay in SK-MES-1 cells, NCI-H520 cells (B), and SK-MES-1 cells transfected with si-LPCRL or si-NC (C), followed by treatment with various concentrations of cisplatin for 48 h. **D** Representative Annexin V-FITC/PI staining images (left panel) showing apoptosis in si-LPCRL- or si-NC-transfected SK-MES-1 cells after 24 h of treatment with 40 µM cisplatin and quantification of apoptotic rates (right panel). **E** Western blot analysis showing the protein expression of γ-H2AX (a DNA damage marker) and full-length/cleaved PARP1 (a marker of apoptosis) in SK-MES-1 cells transfected with si-LPCRL or si-NC following 48 h of 20 µM cisplatin treatment. **F-G** Colony formation (F) and Transwell (G) assays showing the proliferative capacity and migratory capacity of si-LPCRL- or si-NC-transfected SK-MES-1 cells. Representative images (left panel) and quantitative analysis (right panel) are presented. **H** Western blot analysis showing the protein expression of PCNA, N-cadherin, and vimentin in si-LPCRL- or si-NC-transfected SK-MES-1 cells. **I-J** BALB/c nude mice (*n* = 9 per group) were subcutaneously injected with si-LPCRL- or si-NC-transfected SK-MES-1 cells to induce tumor formation. (I) Representative images of excised tumors; (J) tumor growth curves generated from measurements at the indicated time points. **K** Representative immunohistochemical (IHC) images (left panel) and Ki-67 quantification by H-scores (right panel) in the tumors from (I). Scale bars: 100 μm (upper); 50 μm (lower). **L-M** In vivo metastasis was assessed in NOD/SCID mice (*n* = 3 per group) following intravenous injection of luciferase-expressing SK-MES-1 cells transfected with si-LPCRL or si-NC. Representative bioluminescence mouse images (L) and the corresponding quantification of luminescence radiance (M) are shown. **N** Representative H&E-stained images of lung metastatic lesions; red arrows indicate metastatic tumor foci. Scale bars: 500 μm (upper); 100 μm (lower). **O** Statistical analysis of the number of lung metastatic lesions in the indicated groups

To further investigate the role of LPCRL in primary cisplatin resistance in LUSC, we first assessed its expression in SK-MES-1 and NCI-H520 cells. RT‒qPCR analysis revealed significantly higher LPCRL expression in SK-MES-1 cells than in NCI-H520 cells (Fig. 2A). Consistent with these findings, SK-MES-1 cells exhibited greater intrinsic cisplatin resistance, as indicated by a higher IC_50_ in the MTT assays (Fig. 2B). To silence the nucleus-localized LPCRL, we designed a panel of siRNAs, shRNAs, and ASOs and evaluated their silencing efficacy (Supplementary Fig. S2A-S2C). Among these, only one siRNA demonstrated high interference efficiency. This siRNA, which targets the stem region of a predicted stem loop in the LPCRL secondary structure (modeled via RNAstructure software via the minimum free energy method), was selected for subsequent experiments (Supplementary Fig. S2D). We also stably overexpressed exogenous LPCRL in NCI-H520 cells, which presented relatively low endogenous LPCRL levels (Supplementary Fig. S2E). Functionally, LPCRL silencing significantly decreased the IC_50_ of cisplatin in SK-MES-1 cells (Fig. 2C), whereas its overexpression increased the IC_50_ of cisplatin in NCI-H520 cells (Supplementary Fig. S2F). Given that cisplatin exerts its antitumor effects primarily by inducing DNA damage and subsequent apoptosis, we next examined these processes. Flow cytometry analysis revealed that LPCRL silencing enhanced cisplatin-induced apoptosis in SK-MES-1 cells (Fig. 2D), which was supported by elevated levels of γ-H2AX (a DNA damage marker) and cleaved PARP1 (an apoptosis marker; Fig. 2E). Conversely, LPCRL overexpression in NCI-H520 cells attenuated cisplatin-induced apoptosis and reduced the expression of these markers (Supplementary Fig. S2G, S2H). Moreover, consistent with our observations under cisplatin treatment, administration of paclitaxel, another first-line chemotherapeutic agent widely used for LUSC, to LPCRL-knockdown SK-MES-1 cells similarly yielded a reduced IC50 value, enhanced apoptosis, and elevated cleaved PARP1 levels (Supplementary Fig. S2I–S2K).

To investigate the impact of LPCRL on LUSC cell proliferation and migration in vitro, we performed colony formation and Transwell migration assays. LPCRL knockdown significantly suppressed the proliferation and migration of SK-MES-1 cells (Fig. 2F and G), whereas its ectopic overexpression enhanced these abilities in NCI-H520 cells (Supplementary Fig. S2L, S2M). Consistent with these phenotypic alterations, the expression of proliferation markers (PCNA) and epithelial‒mesenchymal transition (EMT)-related migration markers (N-cadherin and vimentin) was downregulated following LPCRL knockdown in SK-MES-1 cells (Fig. 2H) but upregulated upon LPCRL overexpression in NCI-H520 cells (Supplementary Fig. S2N).

To further validate the role of LPCRL in promoting proliferation in vivo, we subcutaneously injected BALB/c nude mice with SK-MES-1 cells transfected with LPCRL-targeting siRNA (si-LPCRL) or control siRNA (si-NC) (Supplementary Fig. S2O). Compared with the si-NC, si-LPCRL not only reduced the tumor incidence but also suppressed the tumor growth rate (Fig. 2I and J). Consistently, immunohistochemical (IHC) analysis revealed that LPCRL knockdown reduced Ki-67 positivity (a marker of cell proliferation; Fig. 2K). To further assess the role of LPCRL in metastasis in vivo, we established an experimental metastasis model by intravenously injecting NOD/SCID mice with luciferase-labeled SK-MES-1 cells transfected with si-NC or si-LPCRL (Supplementary Fig. S2P). si-LPCRL attenuated the metastatic burden, as shown by a reduced bioluminescence signal in the lungs (Fig. 2L and M). Histological examination of lung tissues confirmed a reduction in both the size and number of metastatic lesions in the si-LPCRL group (Fig. 2N and O). Collectively, these findings indicate that LPCRL contribute to cisplatin resistance and promote tumor proliferation and metastasis in LUSC.

### LPCRL binds to MIB1 and inhibits ubiquitin-mediated degradation

**Fig. 3:**
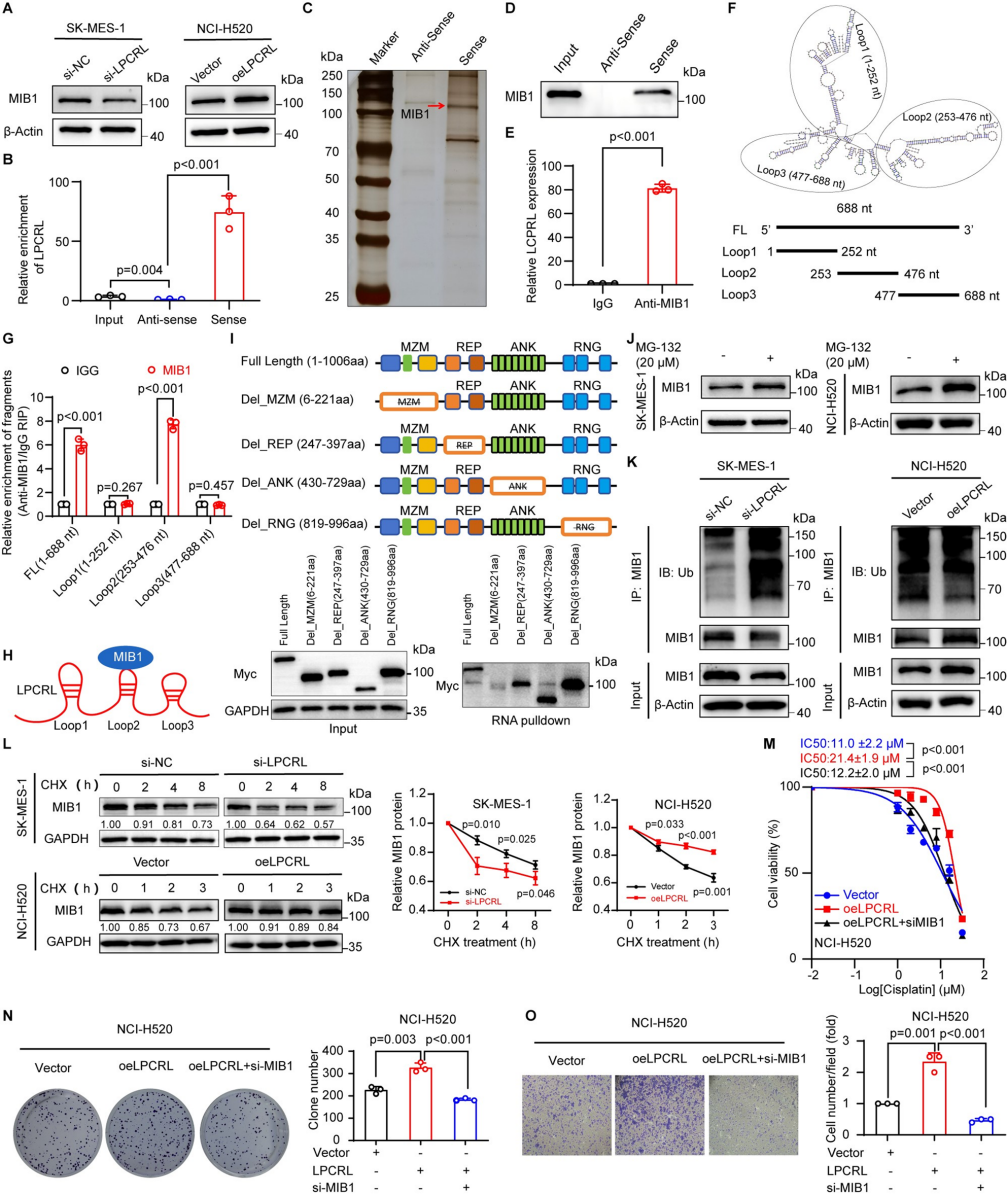
LPCRL binds to MIB1 and blocks its ubiquitination. **A** Western blot analysis of MIB1 protein levels in SK-MES-1 cells (si-LPCRL/si-NC) and NCI-H520 cells (oeLPCRL/vector). **B** RT‒qPCR validation of LPCRL enrichment in SK-MES-1 pull-down assays. **C** Silver staining of proteins pulled down by the biotinylated LPCRL probe in SK-MES-1 cells. **D** MIB1 enrichment in pull-down samples from SK-MES-1 lysates using biotinylated LPCRL RNA. **E** RIP assays with anti-MIB1 antibody showing LPCRL enrichment versus IgG control in SK-MES-1 cells. **F** Schematic representation of the full-length LPCRL and its truncated variants (M1, M2, M3) on the basis of secondary structure prediction. **G** RIP assays in SK-MES-1 cells expressing full-length LPCRL or truncations, showing enrichment of full-length and M2 (253–476 nt). **H** Schematic illustration of the interaction between LPCRL and MIB1. **I** Schematic of MIB1 domain architecture (MZM, REP, ANK, and RNG domains). RNA pull-down and Western blot analyses in 293T cells expressing full-length or truncated MIB1 constructs revealed that deletion of the MZM domain abolished the interaction between MIB1 and LPCRL, whereas truncation of the REP, ANK, or RNG domains had no effect on their interaction. **J** Western blot analysis of MIB1 protein levels in SK-MES-1 and in NCI-H520 cells following 6 h of treatment with the proteasome inhibitor MG132 (20 µM). **K** Western blot analysis of MIB1 ubiquitination levels in SK-MES-1 (si-LPCRL/si-NC) and NCI-H520 (oeLPCRL/vector) cells. **L** Western blot analysis of MIB1 protein levels in SK-MES-1 (si-LPCRL/si-NC) and NCI-H520 (oeLPCRL/vector) cell lines after CHX (50 µg/mL) treatment at indicated times; protein levels were normalized to those of GAPDH via ImageJ. **M** MTT assay for cell viability in NCI-H520 cells (oeLPCRL + si-MIB1) after 48 h cisplatin treatment. **N-O** Colony formation (N) and Transwell assays (O) assessing the proliferative and migratory capacities of NCI-H520 cells (oeLPCRL + si-MIB1). Representative images (left) and quantification of colony numbers or relative migrated cell numbers (right) are shown

Antisense lncRNAs regulate the expression of their corresponding sense genes at the transcriptional or posttranscriptional level through interactions with DNA, RNA, or proteins [12]. Notably, LPCRL is transcribed from an antisense intronic region within the *MIB1* gene. We therefore hypothesized that LPCRL may promote LUSC progression by regulating MIB1, an E3 ubiquitin ligase known to be overexpressed in lung cancer and associated with poor prognosis [26]. Consistent with this hypothesis, CPTAC data and our IHC analyses confirmed significantly elevated MIB1 protein levels in LUSC tissues relative to normal tissues (Supplementary Fig. S3A, S3B). Surprisingly, neither LPCRL knockdown nor LPCRL overexpression significantly altered MIB1 mRNA expression (Supplementary Fig. S3C, S3D); likewise, modulating MIB1 expression did not affect the abundance of LPCRL transcripts (Supplementary Fig. S3E, S3F). However, LPCRL knockdown markedly reduced MIB1 protein levels, whereas LPCRL overexpression significantly increased them (Fig. 3A), indicating a posttranscriptional regulatory mechanism.

Previous studies have shown that lncRNAs can stabilize their interacting proteins by inhibiting ubiquitin-proteasome-mediated degradation [27–30]. To determine if LPCRL physically interacts with MIB1, we first employed RPISeq, which predicts a high-confidence interaction (RF/SVM scores: 0.65/0.81). We next performed RNA pull-down assays in SK-MES-1 cells using a biotin-labeled LPCRL probe. Following efficient probe enrichment (Fig. 3B), silver staining revealed a specific ~ 110 kDa band (Fig. 3C), which was confirmed via western blot analysis as MIB1 (Fig. 3D). RNA immunoprecipitation (RIP) assays independently validated this interaction (Fig. 3E). To map the binding domain, we analyzed the secondary structure of LPCRL, identifying three major loops: Loop1 (1–252 nt), Loop2 (253–476 nt), and Loop3 (477–688 nt) (Fig. 3F). RIP assays with full-length and truncated LPCRL constructs revealed significant enrichment only for the full-length and Loop2 fragments (Fig. 3G), thereby mapping the core MIB1-binding region to nucleotides 253–476 (Fig. 3H). To further define the molecular basis underlying MIB1-LPCRL interaction, we performed domain truncation mapping in a cellular context. MIB1 harbors four conserved domains, namely MZM, REP, ANK, and RNG [31]. RNA pull-down assays using truncated Myc-tagged MIB1 constructs revealed that deletion of the MZM domain, but not REP, ANK, or RNG domains, abolished the binding of MIB1 to LPCRL. These results demonstrate that the MZM domain of MIB1 is indispensable for its direct interaction with LPCRL (Fig. 3I).

To assess whether LPCRL stabilizes MIB1 via the ubiquitin-proteasome pathway, LUSC cells were treated with the proteasome inhibitor MG132 or the protein synthesis inhibitor cycloheximide (CHX). MG132 treatment significantly increased MIB1 protein levels in both SK-MES-1 and NCI-H520 cells (Fig. 3J), confirming the proteasomal degradation of MIB1. Consistent with these findings, LPCRL knockdown increased MIB1 ubiquitination, whereas LPCRL overexpression reduced MIB1 ubiquitination (Fig. 3K). In CHX chase assays, LPCRL silencing accelerated MIB1 degradation, whereas LPCRL overexpression extended its half-life (Fig. 3L). These results demonstrate that LPCRL stabilizes MIB1 by inhibiting its ubiquitin-mediated proteasomal degradation.

To investigate whether LPCRL promotes LUSC progression via MIB1, we first examined the role of MIB1 in LUSC cells. In SK-MES-1 cells, MIB1 knockdown significantly downregulated key markers of cell proliferation and migration, including PCNA, N-cadherin, and vimentin (Supplementary Fig. S3G). Furthermore, MIB1 silencing followed by cisplatin treatment markedly increased the levels of γ-H2AX and cleaved PARP, indicating enhanced DNA damage and apoptosis (Supplementary Fig. S3H). To confirm that LPCRL functions through MIB1, we silenced MIB1 in NCI-H520 cells stably overexpressing LPCRL. MTT assays revealed that MIB1 knockdown abolished the LPCRL-induced increase in the IC_50_ of cisplatin (Fig. 3M). Furthermore, colony formation and Transwell assays revealed that MIB1 silencing attenuated the enhanced proliferative and migratory capacities conferred by LPCRL overexpression (Fig. 3N and O), which was consistent with decreased PCNA and vimentin protein levels (Supplementary Fig. S3I). Upon cisplatin exposure, MIB1 silencing abrogated the LPCRL-mediated suppression of γ-H2AX and cleaved PARP (Supplementary Fig. S3J). These findings collectively support that MIB1 is a key mediator of the oncogenic effects of LPCRL in regulating proliferation, migration, and cisplatin resistance in LUSC.

### LPCRL stabilizes MIB1 via USP15-mediated deubiquitination

**Fig. 4:**
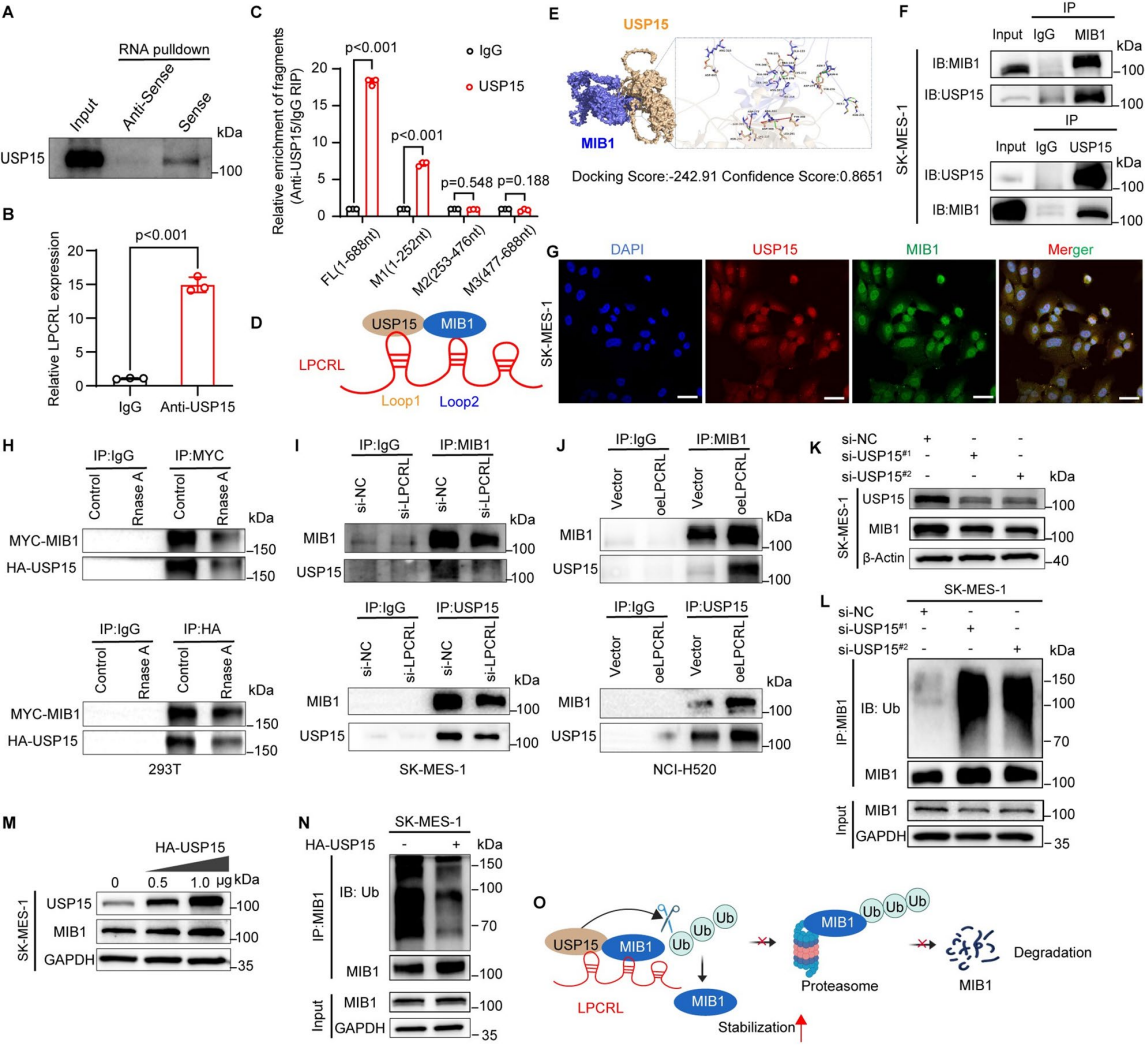
LPCRL serves as a scaffold that promotes the interaction between MIB1 and USP15. **A** Western blot analysis of USP15 enrichment in pull-down samples from SK-MES-1 cell lysates via biotinylated LPCRL RNA. **B** RIP assays with an anti-USP15 antibody in SK-MES-1 cells showing LPCRL enrichment relative to the IgG control group. **C** RIP assays with an anti-USP15 antibody in SK-MES-1 cells (transfected with plasmids expressing full-length LPCRL or the indicated truncation mutants) showing enrichment of the full-length LPCRL and the M1 truncation (1–252 nt). **D** Schematic illustration of the interaction between LPCRL, MIB1, and USP15. **E** Predicted binding mode between MIB1 (blue) and USP15 (wheat) via the protein‒protein docking program HDock. **F** Co-IP showing the endogenous interaction between MIB1 and USP15 in SK-MES-1 cells. **G** Representative immunofluorescence images of endogenous MIB1 (green) and USP15 (red) in SK-MES-1 cells; nuclei were stained with DAPI (blue). Scale bar: 20 μm. **H** Co-IP showing the interaction between Myc-MIB1 and HA-USP15 in lysates from HEK293T cells transiently cotransfected with Myc-MIB1 and HA-USP15 with or without RNase A treatment. **I–J** Co-IP showing the endogenous interaction between MIB1 and USP15 in SK-MES-1 cells with LPCRL knockdown (I) or in NCI-H520 cells with stable LPCRL overexpression (oeLPCRL) (J). **K–L** Western blot analysis of MIB1 protein levels (K) and its ubiquitination (L) in SK-MES-1 cells transfected with si-USP15 or siNC. **M–N** Western blot analysis of MIB1 protein levels (M) and its ubiquitination (N) in SK-MES-1 cells transfected with USP15-expressing plasmids. **O** Schematic illustration showing that LPCRL acts as a molecular scaffold to facilitate the interaction between MIB1 and USP15, enabling USP15-mediated deubiquitination of MIB1, thereby preventing its proteasomal degradation and enhancing protein stability

LPCRL lacks protein-coding capacity; therefore, the precise mechanism by which it interacts with MIB1 to regulate MIB1 ubiquitination remains incompletely understood. Accumulating evidence indicates that lncRNAs function as molecular scaffolds to facilitate protein‒protein interactions [32–34]. On the basis of these findings, we hypothesized that LPCRL may act as a scaffold to recruit a deubiquitinating enzyme that interacts with MIB1, thereby inhibiting MIB1 ubiquitination and subsequent proteasomal degradation. To identify MIB1-associated deubiquitinating enzymes, we analyzed BioID proteomic datasets and BioGRID interactors, which identified USP9X, CYLD, and USP15 as potential MIB1-interacting candidates [35]. While interactions between MIB1 and USP9X or CYLD have been reported [36, 37], the MIB1-USP15 interaction remains uncharacterized. USP15 is a critical deubiquitinating enzyme implicated in multiple malignancies and has been reported to promote lung cancer cell proliferation [38]. Moreover, RPISeq analysis predicted a high-probability interaction between LPCRL and USP15 (RF/SVM scores: 0.75 and 0.97). On the basis of these findings, we hypothesized that LPCRL serves as a molecular scaffold, facilitating the interaction of MIB1 with USP15 in LUSC cells. Western blot analysis confirmed the specific enrichment of USP15 by the biotin-labeled LPCRL probe (Fig. 4A). Furthermore, RIP assays revealed significant enrichment of LPCRL in USP15-immunoprecipitated complexes (Fig. 4B). Similarly, using full-length and truncated LPCRL constructs transfected into SK-MES-1 cells, RIP assays revealed significant enrichment of the Loop1 region (nucleotides 1–252) in USP15-immunoprecipitated complexes (Fig. 4C). Collectively, these results indicate that MIB1 primarily binds to Loop2 of LPCRL, whereas USP15 preferentially associates with Loop1, supporting the role of LPCRL as a molecular scaffold (Fig. 4D).

These results indicate that LPCRL binds independently to MIB1 and USP15. We therefore hypothesized that LPCRL serves as a molecular scaffold to promote the MIB1-USP15 interaction. To this end, we first assessed whether MIB1 and USP15 physically interact. Molecular docking analysis predicted multiple potential binding modes between MIB1 and USP15 (Fig. 4E). Co-IP assays confirmed the endogenous interaction between MIB1 and USP15 in both SK-MES-1 and NCI-H520 cells (Fig. 4F, Supplementary Fig. S4A). Furthermore, immunofluorescence (IF) staining demonstrated their nuclear colocalization in these cell lines (Fig. 4G, Supplementary Fig. S4B), which is consistent with the established nuclear localization of LPCRL. To further validate the scaffolding role of LPCRL, we initially conducted Co-IP assays to assess exogenous MIB1-USP15 interactions in HEK293T cells with or without RNase A treatment. The results showed that MIB1 interacted with USP15 in the absence of RNase A; however, this interaction was markedly diminished upon RNase A treatment (Fig. 4H). Additionally, LPCRL knockdown weakened the interaction between MIB1 and USP15 (Fig. 4I), whereas LPCRL overexpression increased this interaction (Fig. 4J), thus supporting the hypothesis that LPCRL is essential for mediating this interaction. Taken together, these data indicate that LPCRL functions as a molecular scaffold to facilitate the formation of the MIB1-USP15 complex.

Functionally, USP15 knockdown reduced MIB1 protein levels and increased MIB1 ubiquitination (Fig. 4K and L; Supplementary Fig. S4C, S4D). Conversely, USP15 overexpression had the opposite effect (Fig. 4M and N; Supplementary Fig. S4E, S4F). Moreover, LPCRL knockdown decreased the USP15 protein level without affecting its mRNA level, whereas LPCRL overexpression increased it (Supplementary Fig. S4G-S4J). This finding suggests that LPCRL posttranslationally stabilizes both MIB1 and USP15. Collectively, these findings demonstrate that LPCRL promotes the assembly of the USP15-MIB1-LPCRL ternary complex, which enables USP15-mediated deubiquitination of MIB1, thereby shielding it from proteasomal degradation and enhancing its protein stability (Fig. 4O).

### The LPCRL-MIB1-USP15 complex activates the Notch signaling pathway

**Fig. 5:**
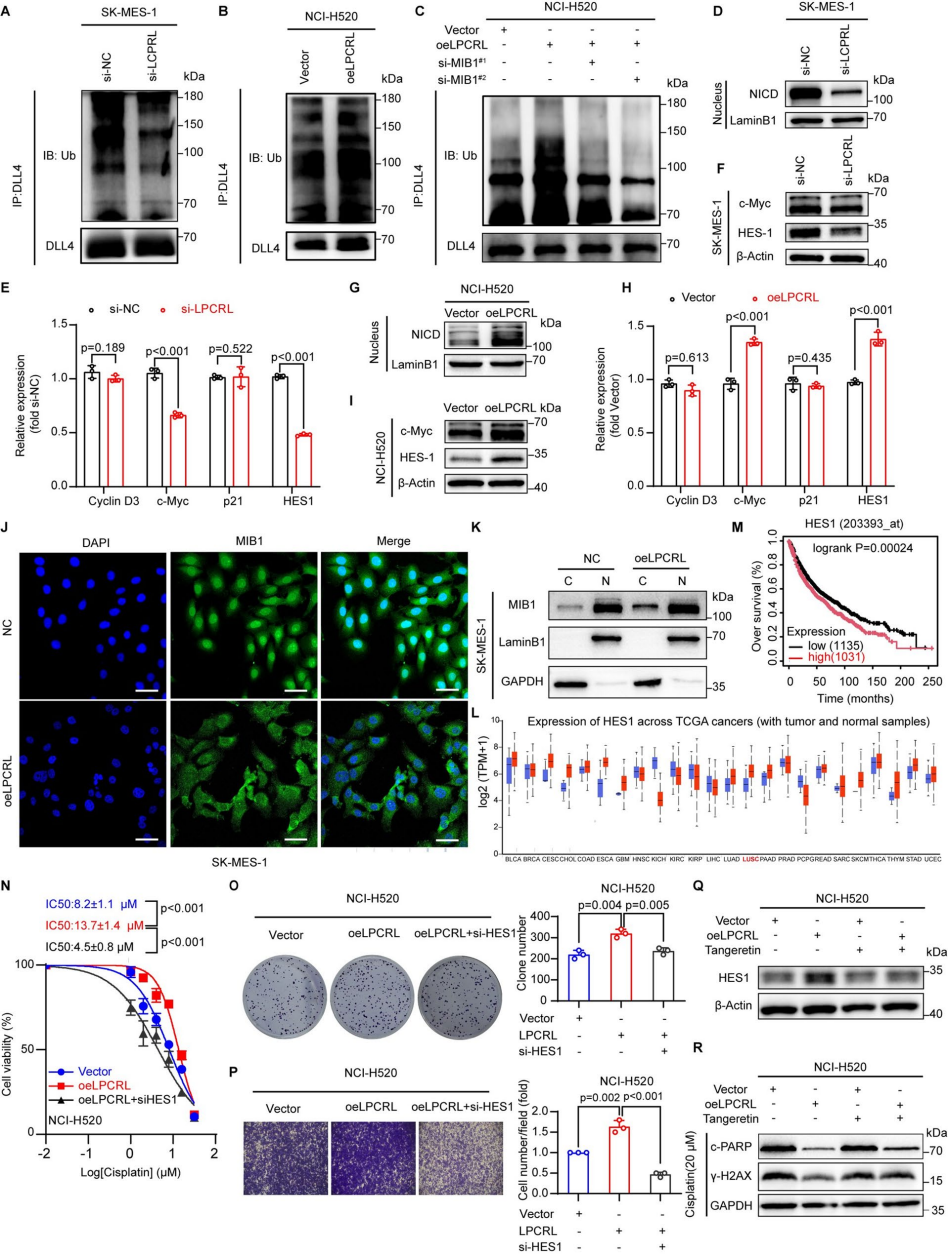
The LPCRL-MIB1-USP15 complex activates the Notch signaling pathway. **A-B** Co-IP analysis of DLL4 ubiquitination levels in SK-MES-1 cells (si-LPCRL/si-NC) (A) and in NCI-H520 cells (oeLPCRL/vector) (B). **C** Co-IP analysis of DLL4 ubiquitination in NCI-H520 cells (oeLPCRL + si-MIB1). **D**,** G** Western blot analysis of nuclear NICD protein levels in SK-MES-1 cells (si-LPCRL/si-NC) (D) and in NCI-H520 cells (oeLPCRL/vector) (G). **E**,** H** RT‒qPCR analysis of Notch target genes (HES1, p21, c-Myc, and CyclinD1) in SK-MES-1 cells (si-LPCRL/si-NC) (E) and in NCI-H520 cells (oeLPCRL/vector) (H). **F**,** I** Western blot analysis of c-Myc and HES1 protein levels in SK-MES-1 cells (si-LPCRL/si-NC) (F) and in NCI-H520 cells (oeLPCRL/vector) (I). **J** Representative immunofluorescence images showing the subcellular localization of MIB1 (green) in SK-MES-1 cells with or without transient LPCRL overexpression; nuclei were counterstained with DAPI (blue). Scale bar: 20 μm. **K** Western blot analysis of MIB1 protein levels in subcellular fractions from SK-MES-1 cells with or without transient LPCRL overexpression. C, cytosolic fraction; N, nuclear fraction. **L** Bar chart showing HES1 expression levels across various TCGA cancer types (tumor vs. normal samples). **M** Kaplan‒Meier analysis showing that LUSC patients with higher HES1 expression had worse overall survival (OS). **N** Cell viability was evaluated via an MTT assay in NCI-H520 cells (oeLPCRL + si-HES1) following 48 h of treatment with the indicated concentrations of cisplatin. **O-P** Colony formation (O) and Transwell assays (P) assessing the proliferative and migratory capacities of NCI-H520 cells (oeLPCRL + si-HES1). Representative images (left) and quantification of colony numbers or relative migrated cell numbers (right) are shown. **Q** Western blot analysis of HES1 protein levels in NCI-H520 cells stably overexpressing LPCRL (oeLPCRL) with or without treatment with tangeretin (a Notch inhibitor, 20 µM). **R** Western blot analysis of γ-H2AX and c-PARP protein levels in LPCRL-overexpressing NCI-H520 cells treated with cisplatin (20 µM) in the presence or absence of tangeretin (20 µM)

Our previous studies revealed that the LPCRL-MIB1-USP15 complex enhances MIB1 stability in LUSC cells. As an E3 ubiquitin ligase, MIB1 activates the Notch signaling pathway via ubiquitination and endocytosis of the intracellular domains of its ligands (Delta/Jagged). Notably, activation of the Notch signaling pathway is critical for tumorigenesis and progression and is implicated in promoting proliferation, metastasis, and chemoresistance in various cancers [39, 40]. We thus assessed whether LPCRL-driven malignant progression in LUSC cells is dependent on the Notch signaling pathway. Co-IP assays demonstrated that MIB1 interacts with DLL4, a Notch ligand, in both SK-MES-1 and NCI-H520 cells (Supplementary Fig. S5A, S5B). LPCRL knockdown significantly reduced DLL4 ubiquitination (Fig. 5A), whereas LPCRL overexpression increased it (Fig. 5B). Notably, this increase was substantially mitigated upon MIB1 knockdown (Fig. 5C), confirming the role of MIB1 in this process. Furthermore, LPCRL knockdown decreased the nuclear accumulation of the Notch intracellular domain (NICD) (Fig. 5D) and downregulated the mRNA and protein levels of the Notch downstream effectors HES1 and c-Myc (Fig. 5E and F). Conversely, LPCRL overexpression upregulated these effectors (Fig. 5G and I), indicating that LPCRL effectively activated the Notch signaling pathway.

Our experiments demonstrated that LPCRL inhibits MIB1 degradation via the USP15-mediated ubiquitin–proteasome pathway in LUSC cell nuclei, thereby maintaining MIB1 homeostasis. However, MIB1 is known to activate the Notch signaling pathway by catalyzing the ubiquitination of its specific ligands in the cytoplasm [31]. We thus investigated whether the LPCRL-MIB1-USP15 complex facilitates MIB1 nuclear export to enable Notch signaling pathway activation. Both nuclear-cytoplasmic fractionation and IF assays revealed that the overexpression of LPCRL or USP15 increased the cytoplasmic MIB1 level (Fig. 5J and K; Supplementary Fig. S5C, S5D). Collectively, these findings indicate that the LPCRL-MIB1-USP15 complex regulates not only MIB1 stability but also its nuclear export, providing new insights into the molecular mechanism of LPCRL in LUSC.

HES1 plays a pivotal role in tumorigenesis, tumor progression, and therapeutic resistance [41]. To determine whether LPCRL contributes to LUSC progression by modulating HES1, we first assessed the role of HES1 in LUSC via multiple approaches. TCGA database analysis revealed significantly elevated HES1 mRNA in LUSC tissues compared with normal lung tissues (Fig. 5L), and Kaplan‒Meier survival analysis revealed that high HES1 expression was correlated with reduced overall survival in LUSC patients (Fig. 5M). CPTAC database data and our IHC analysis confirmed that HES1 protein expression was increased in LUSC clinical samples compared with normal tissues (Supplementary Fig. S5E, S5F). Furthermore, HES1 silencing reduced the expression of proliferation- and migration-related proteins (PCNA, N-cadherin, and vimentin; Supplementary Fig. S5G) and elevated cisplatin-induced γ-H2AX and cleaved PARP levels (Supplementary Fig. S5H). HES1 expression was also elevated in cisplatin-resistant patient-derived xenograft (PDX) tissues (Supplementary Fig. S5I).

NCI-H520 cells stably overexpressing LPCRL were subsequently transfected with HES1-targeting siRNAs. MTT assays revealed that HES1 knockdown reversed the increase in the IC_50_ of cisplatin induced by LPCRL (Fig. 5N) and abolished the suppressive effect of LPCRL on cisplatin-induced γ-H2AX and cleaved PARP (Supplementary Fig. S5J). Colony formation and Transwell migration assays further demonstrated that HES1 silencing mitigated the increase in cell proliferation and migration driven by LPCRL (Fig. 5O and P). Consistent with these functional changes, HES1 knockdown also reduced the expression of PCNA and vimentin (Supplementary Fig. S5K). Notably, treatment with tangeretin, a Notch inhibitor, also inhibited LPCRL-mediated HES1 upregulation and cisplatin resistance. This was evidenced by the reversal of the effects of LPCRL on γ-H2AX and cleaved PARP (c-PARP) levels (Fig. 5Q and R). Collectively, these findings confirm that LPCRL exerts its oncogenic functions through the MIB1-USP15-Notch-HES1 axis.

### LPCRL is potential therapeutic targets for the treatment of LUSC

To assess the therapeutic potential of LPCRL inhibition, we knocked down LPCRL in primary LUSC cells derived from a patient. LPCRL silencing significantly suppressed colony formation, reduced the cisplatin IC_50_, and enhanced apoptosis, as evidenced by elevated γ-H2AX and c-PARP levels (Fig. 1K and L; Supplementary Fig. S6A, S6B). MIB1 protein expression also decreased without altering its mRNA levels (Supplementary Fig. S6C, S6D). The endogenous interaction between MIB1 and USP15 was confirmed by Co-IP, and manipulation of USP15 levels altered MIB1 protein expression accordingly (Supplementary Fig. S6E, S6F). Collectively, these results corroborate our previous observations in LUSC cell lines and support the therapeutic potential of targeting LPCRL in LUSC.

**Fig. 6:**
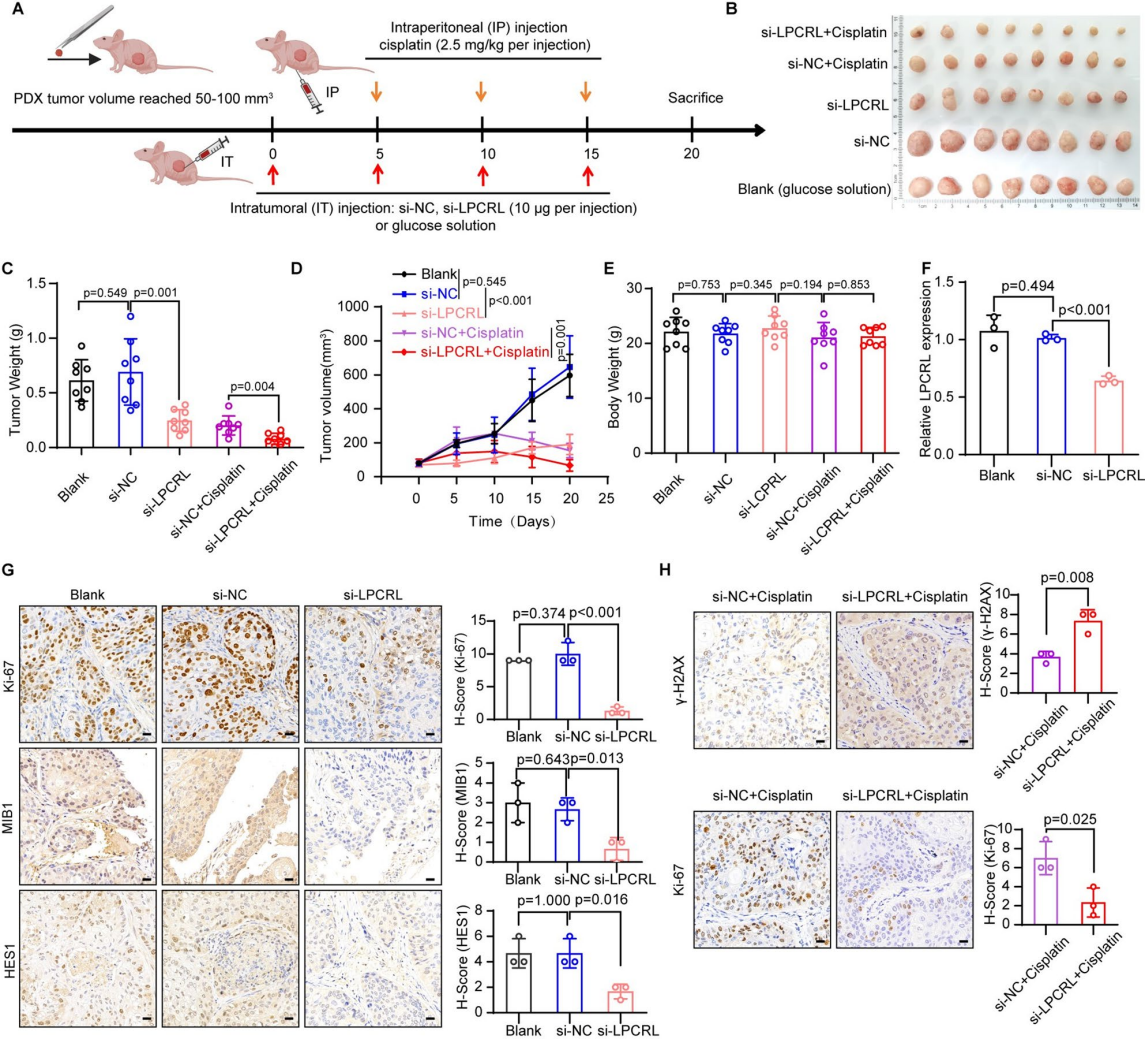
Silencing of LPCRL inhibits tumor growth and enhances cisplatin sensitivity in PDX models. **A** Schematic illustration of PDX model establishment, followed by the administration of LPCRL-targeting siRNA (si-LPCRL, 10 µg per injection), si-NC, glucose solution (Blank), si-NC plus cisplatin (2.5 mg/kg per injection), or si-LPCRL plus cisplatin. Nude mice were euthanized after the indicated treatments were completed. **B-C** Excised tumors were imaged (B), with tumor weights measured in each group (C). **D** Growth curves of subcutaneously transplanted tumors across groups. **E** Body weights were measured in each group. **F** RT‒qPCR analysis of LPCRL expression in the blank, si-NC, and si-LPCRL groups. **G** Representative immunohistochemistry (IHC) images (left) and quantification (H-score values, right) of Ki-67, MIB1, and HES1 in tumors from the glucose solution (Blank), si-NC, and si-LPCRL groups. Scale bar: 20 μm. **H** Representative IHC images (left) and quantification (H-score values, right) of Ki-67 and γ-H2AX in tumors from the si-NC plus cisplatin and si-LPCRL plus cisplatin groups. Scale bar: 20 μm

To test its efficacy in vivo, we further employed a PDX model established from the same LUSC patient as the primary cells. When the tumor volume reached 50–100 mm³, the mice were intratumorally injected with LPCRL-targeting siRNA (si-LPCRL), si-NC, or glucose solution (Blank). Cisplatin was administered intraperitoneally every five days (Fig. 6A). Consistent with the in vitro findings, si-LPCRL significantly inhibited tumor growth and reduced tumor weight compared with those in the si-NC and blank groups. Importantly, the combination of cisplatin and si-LPCRL enhanced the inhibitory effect compared with that of cisplatin plus si-NC (Fig. 6B and D). No significant body weight changes were observed across groups (Fig. 6E), indicating that si-LPCRL inhibits tumor growth and enhances cisplatin sensitivity without overt systemic toxicity in LUSC. The knockdown efficiency was confirmed by RT‒qPCR (Fig. 6F). Compared with those in the si-NC or blank groups, IHC staining revealed markedly lower Ki67, MIB1, and HES1 expression in the si-LPCRL-treated tumors (Fig. 6G), and the si-LPCRL plus cisplatin combination notably increased DNA damage (elevated γ-H2AX) and inhibited proliferation (decreased Ki67) compared with those in the cisplatin plus si-NC group (Fig. 6H). These results demonstrate that targeting LPCRL suppresses cancer cell proliferation, reduces MIB1 and its downstream HES1 expression, and enhances cisplatin sensitivity in vivo, supporting its potential as a therapeutic target.

## Discussion

Platinum-based chemotherapy, particularly cisplatin, is a cornerstone of LUSC treatment; however, its efficacy is severely hindered by the emergence of drug resistance [42]. These challenges underscore the urgent need to elucidate the molecular mechanisms underlying cisplatin resistance and identify novel therapeutic strategies. In this study, we identified a novel antisense lncRNA, designated LPCRL, which is highly expressed in primary cisplatin-resistant LUSC PDX tissues. Functional and mechanistic studies revealed that LPCRL acts as a molecular scaffold to facilitate the assembly of the USP15/MIB1 complex, thereby activating the Notch signaling pathway and promoting primary cisplatin resistance and tumor progression in LUSC. Our findings uncover a novel LPCRL/USP15/MIB1/Notch regulatory axis and highlight LPCRL as a promising therapeutic target for overcoming cisplatin resistance in LUSC.

A key finding of our study is the identification of LPCRL as a novel oncogenic antisense lncRNA that is tightly associated with primary cisplatin resistance in LUSC. LPCRL is transcribed from the MIR133A1HG gene locus on chromosome 18q11.2, which overlaps the antisense strand of the twelfth intron of MIB1. The MIR133A1HG locus is well known as a precursor for the tumor-suppressive microRNAs miR-133a and miR-1, which are frequently downregulated in various solid tumors and exert anti-tumor effects by inhibiting cell proliferation and metastasis [43, 44]. Interestingly, MIR133A1HG has also been implicated in hematological malignancies, with higher expression correlating with better prognosis in acute myeloid leukemia patients [45]. In contrast, our work revealed that LPCRL, a lncRNA isoform from the same locus, functions as an oncogene in LUSC. This functional divergence underscores the remarkable transcriptional complexity of a single genomic locus and highlights the critical importance of isoform-specific investigations in cancer research.

Accumulating evidence indicates that lncRNAs can function as molecular scaffolds to facilitate the assembly of protein complexes, thereby regulating various biological processes [32, 33]. In this study, we demonstrated that LPCRL acts as a molecular scaffold to directly mediate the physical interaction between MIB1 and USP15 in the nucleus of LUSC cells. MIB1 is an E3 ubiquitin ligase that is overexpressed in lung cancer and associated with poor prognosis [26], while USP15 is a deubiquitinating enzyme (DUB) that has been implicated in multiple malignancies and promotes lung cancer cell proliferation [38]. Our study revealed that LPCRL binds to MIB1 and USP15 through distinct regions: MIB1 primarily binds to Loop2 (253–476 nt) of LPCRL, whereas USP15 preferentially associates with Loop1 (1–252 nt). This specific binding pattern enables LPCRL to serve as a bridge to bring MIB1 and USP15 into close proximity, facilitating the formation of the USP15-MIB1-LPCRL ternary complex. Notably, our initial bioinformatic screening identified USP9X and CYLD as additional potential MIB1-interacting deubiquitinases (DUBs). USP9X, a key member of the USP family, has been reported to undergo S-nitrosylation and subsequently deubiquitinate and stabilize MIB1, thereby promoting Notch1 activation in the pathogenesis of calcific aortic valve disease [37]. This further supports the notion that MIB1 is tightly controlled by multiple DUBs. In the present study, we focused on USP15 to reveal its previously uncharacterized role in regulating MIB1 in LUSC and cisplatin resistance. However, we do not exclude the possibility that USP9X may also modulate MIB1 stability and downstream Notch signaling in LUSC. Future studies will investigate whether USP9X and USP15 function coordinately to regulate MIB1 ubiquitination and protein levels, thereby synergistically affecting Notch pathway activation and cisplatin response.

The formation of the LPCRL/USP15/MIB1 complex plays a pivotal role in regulating MIB1 stability and subcellular localization in LUSC cells. Our mechanistic studies demonstrated that USP15, as a component of this complex, mediates the deubiquitination of MIB1, thereby inhibiting its ubiquitin–proteasome-mediated degradation and enhancing its protein stability. Additionally, we found that the LPCRL/USP15/MIB1 complex promotes the nuclear export of MIB1, leading to its cytoplasmic accumulation in LUSC cells. MIB1 is known to activate the Notch signaling pathway by catalyzing the ubiquitination of Notch ligands (e.g., DLL4) in the cytoplasm. Consistent with this, our study showed that the cytoplasmic accumulation of MIB1 induced by LPCRL/USP15, subsequent activation of the Notch signaling pathway, and upregulation of the downstream effector HES1. Notably, the nuclear localization of LPCRL, MIB1, and USP15 and their subsequent regulation of MIB1 nuclear export represent a novel regulatory mechanism for Notch signaling activation. This finding is analogous to the USP7-mediated regulation of PTEN, where nuclear stabilization of PTEN is followed by cytoplasmic translocation to exert its tumor-suppressive effects [46], suggesting that nuclear stabilization and subsequent cytoplasmic translocation may be a common strategy for regulating key signaling proteins in cancer.

From a translational perspective, our study highlighted the potential of LPCRL as a novel therapeutic target for overcoming cisplatin resistance in LUSC. We found that intratumoral administration of LPCRL-targeting siRNA significantly suppresses tumor growth and enhances cisplatin sensitivity in LUSC PDX models, without overt systemic toxicity. The development of effective siRNA-based therapeutics against lncRNAs is often challenging due to their nuclear localization and complex secondary structures. In this study, we successfully identified a specific and efficient siRNA against LPCRL by targeting the stem region of a predicted stem loop in its secondary structure. This finding underscores the importance of considering RNA secondary structure in the design of lncRNA-targeting therapeutics. Recent advancements in structural probing techniques have revealed critical structural motifs and RNA–protein interfaces that contribute to lncRNA dysfunction, providing new opportunities for the development of small molecules, antisense oligonucleotides, and peptidomimetic-based therapeutic agents targeting lncRNAs [47]. Furthermore, with the development of novel drug delivery systems, such as inhalable lipid nanoparticles that enable targeted lung delivery with increased local accumulation and reduced systemic exposure, LPCRL-targeting siRNA holds significant promise for clinical translation as a novel therapeutic strategy for LUSC [48]. Moreover, our preliminary data indicated that LPCRL knockdown also enhances sensitivity to paclitaxel (Supplementary Fig. S2I–S2K), suggesting that the role of LPCRL in chemoresistance may extend beyond cisplatin and support its broader relevance as a therapeutic target in LUSC.

Despite these significant findings, our study has several limitations that warrant further investigation. First, the sample size in our study is relatively small, which may limit the statistical power and generalizability of our findings. Therefore, large-scale, multicenter clinical studies are warranted to validate the association between LPCRL expression and cisplatin response, as well as its prognostic value in patients with LUSC. Second, the nuclear function of MIB1 remains incompletely understood; it may have nuclear substrates beyond Notch ligands, and future studies employing proximity-labeling techniques such as BioID could help delineate the nuclear interactome of MIB1 and uncover its noncanonical roles. Third, the precise mechanisms by which the LPCRL/USP15 complex mediates nuclear export of MIB1 require further elucidation — for instance, through single-cell live-cell imaging and identification of the nuclear export signals (NES) within MIB1 and their regulatory factors. Fourth, although intratumoral injection of LPCRL-targeting siRNA showed promising therapeutic efficacy in our PDX models, this route of administration is not directly translatable to clinical practice. A considerable gap remains between the current delivery strategy and the requirements for safe and effective systemic administration in patients. Developing more efficient and clinically applicable delivery systems is thus essential to facilitate the clinical translation of LPCRL-targeted therapy.

## Conclusions

Our study identified LPCRL as a novel antisense lncRNA that promotes primary cisplatin resistance and tumor progression in LUSC. Mechanistically, LPCRL functions as a molecular scaffold to facilitate the assembly of the USP15/MIB1 complex, which mediates the deubiquitination and stabilization of MIB1 and promotes its nuclear export. The cytoplasmic accumulation of MIB1 leads to activation of the Notch/HES1 signaling pathway, thereby promoting cisplatin resistance and malignant progression in LUSC. Our findings uncover a novel LPCRL/USP15/MIB1/Notch regulatory axis and fill a critical knowledge gap in the molecular regulation of cisplatin resistance in LUSC. LPCRL is highlighted as a promising therapeutic target for overcoming chemotherapy resistance, and future studies focusing on the detailed mechanisms of MIB1 nuclear export, the development of more efficient LPCRL-targeting therapeutics, and the validation of clinical relevance will further advance the translation of these findings into clinical practice for the treatment of LUSC patients.

## Supplementary Information

Supplementary Material 1.

## Data Availability

All raw lncRNA microarray data are available upon request from the corresponding author.

## Abbreviations

LUSC: Lung squamous cell carcinoma
LncRNAs: Long noncoding RNAs
NATs: Natural antisense transcripts
PDX: Patient-derived xenograft
LPCRL: LUSC primary cisplatin resistance-associated lncRNA
CNVs: Copy number variations
Co-IP: Coimmunoprecipitation
FISH: Fluorescence in situ hybridization
RIP: RNA immunoprecipitation
IHC: Immunohistochemistry
