# Supplementary material for: A novel antisense lncRNA, LPCRL, functions as a molecular scaffold for the USP15/MIB1 complex to promote primary cisplatin resistance and tumor progression in lung squamous cell carcinoma
Source: J Exp Clin Cancer Res. 2026 May 1;45:136. doi: 10.1186/s13046-026-03721-7 (PMC13276959; doi:10.1186/s13046-026-03721-7)
Supplement: Supplementary file 1 — Supplementary Material 1. [file 13046_2026_3721_MOESM1_ESM.docx]

Supplemental information for

**A novel antisense lncRNA, LPCRL, functions as a molecular scaffold for the USP15/MIB1 complex to promote primary cisplatin resistance and tumor progression in lung squamous cell carcinoma**

Peng Luo^1,2^, Dapeng Lu^1,2^, Shuang Zhang^1,2^, Wenqian Dong^1,2^, Kai Fang^1,2^, Shihao Yu^1,2^, Bing He^1,2^, Maoxin Zhu^1,2^, Yuee Wang^3^, Xianliang Jiang^4^, Baolong Wang^1,2*^

This file includes:
Supplementary Tables (Table S1 and Table S2)
Supplementary Figures (Figure S1-Figure S6)

Table S1**. The sequences of primers used in this study.**

| Target | Primer sequence (5’-3’) |
| --- | --- |
| *LPCRL* | F: AAGATGAGATGGCTGCTGTTG |
|  | R: CTTATGGTGCTCTGTTACCTGTAA |
| *GAPDH* | F: GGAGCGAGATCCCTCCAAAAT |
|  | R: GGCTGTTGTCATACTTCTCATGG |
| *U6* | F: GCTTCGGCAGCACATATACTAAAAT |
|  | R: CGCTTCACGAATTTGCGTGTCAT |
| *18S* | F: CAGCCACCCGAGATTGAGCA |
|  | R:TAGTAGCGACGGGCGGTGTG |
| *MIB1* | F: CGAGGAGGTGGTGGTAGTGT |
|  | R: GATGCGGAGGTCGTAAGC |
| *USP15* | F: TCAAAATGTGTATCCTGGACCCA |
|  | R: GTGCTATTGGCTCTTGACCTT |
| *HES1* | F: CATTCCAAGCTGGAGAAGGC |
|  | R: GTCACCTCGTTCATGCACTC |
| *p21* | F: TGTCCGTCAGAACCCATGC |
|  | R: AAAGTCGAAGTTCCATCGCTC |
| *Cyclin D3* | F: TACCCGCCATCCATGATCG |
|  | R: AGGCAGTCCACTTCAGTGC |
| *C-MYC* | F: GGCTCCTGGCAAAAGGTCA |
|  | R: CTGCGTAGTTGTGCTGATGT |
| *LPCRL-M1* | F: TCTGCAACTTTTTCCCCTCTTCT |
|  | R: AGTGTTGGCCATTTGATTTGGA |
| *LPCRL-M2* | F: AGGTGAGGTTCTGTGTTCACT |
|  | R:TCACATCAACAGCAGCCATCT |
| *LPCRL-M3* | F: AGCACCATAAGGCCTGTACTT |
|  | R: AGTTACTGCCATAGTATCTGAACA |
| 3' RACE adaptor | GCGCTGACAACGCTCCCGCTGAATTGGAATTTTTTTTTTTTTTTTTTTT |
| 3' RACE outer F | CTGCTGTTGATGTGATTCTC |
| 3' RACE outer R | GCGCTGACAACGCTCCCGCT |
| 3' RACE inner F | CTGCATGACACTAAGCTTAC |
| 3' RACE inner R | CGCTCCCGCTGAATTGGAAT |
| 5' RACE RT | ACAGCAGCCATCTCATCTTG |
| 5' RACE GSP1 | CACTCTATTTGCTGATTCAGC |
| 5' RACE UPM1 | TCTAATACGACTCACTATAGGGCAAGCAGTGGTATCAACGCAGAGTTTTTTTTTTTTTTTTTT |
| 5' RACE GSP2 | TCAGTGAACACAGAACCTCA |
| 5' RACE UPM2 | CTAATACGACTCACTATAGGGC |

Table S2**. The sequences of siRNAs and shRNAs used in this study.**

| Target | sequence (5’-3’) |
| --- | --- |
| si-NC | UUCUCCGAACGUGUCACGUTT |
|  | ACGUGACACGUUCGGAGAATT |
| si-LPCRL-1 | F: UCAAAUGGCCAACACUGAUTT |
|  | R: AUCAGUGUUGGCCAUUUGATT |
| si-LPCRL-2 | F: GCUUACAGGUAACAGAGCATT |
|  | R: UGCUCUGUUACCUGUAAGCTT |
| si-LPCRL-3 | F: CCAUAGCCUAAUUUCACAATT |
|  | R: UUGUGAAAUUAGGCUAUGGTT |
| sh-NC | Top strand:  GATCCGTTCTCCGAACGTGTCACGTAATTCAAGAGATTACGTGACACGTTCGGAGAATTTTTTC |
|  | Bottom strand:  AATTGAAAAAATTCTCCGAACGTGTCACGTAATCTCTTGAATTACGTGACACGTTCGGAGAACG |
| sh-LPCRL-1 | Top strand: GATCCGGACGGCAATGAAGAAGATTTCAAGAGAATCTTCTTCATTGCCGTCCTTTTTTG |
|  | Bottom strand:  AATTCAAAAAAGGACGGCAATGAAGAAGATTCTCTTGAAATCTTCTTCATTGCCGTCCG |
| sh-LPCRL-2 | Top strand:  GATCCGCTTACAGGTAACAGAGCATTCAAGAGATGCTCTGTTACCTGTAAGCTTTTTTG |
|  | Bottom strand:  AATTCAAAAAAGCTTACAGGTAACAGAGCATCTCTTGAATGCTCTGTTACCTGTAAGCG |
| si-MIB1-1 | F: GCUUCCAACUUUAGGUAAATT |
|  | R: UUUACCUAAAGUUGGAAGCTT |
| si-MIB1-2 | F: GCUUGUGAGAACUGUGCUATT |
|  | R: UAGCACAGUUCUCACAAGCTT |
| si-USP15-1 | F: GAAUUGGAUUACAUACUGUTT |
|  | R: ACAGUAUGUAAUCCAAUUCTT |
| si-USP15-2 | F: GUAUAUCUCACAGAAUUGATT |
|  | R: UCAAUUCUGUGAGAUAUACTT |
| si-HES1-1 | F: AGAUCAAUGCCAUGACCUATT |
|  | R: UAGGUCAUGGCAUUGAUCUTT |
| si-HES1-2 | F: GGACAUUCUGGAAAUGACATT |
|  | R: UGUCAUUUCCAGAAUGUCCTT |

### ****
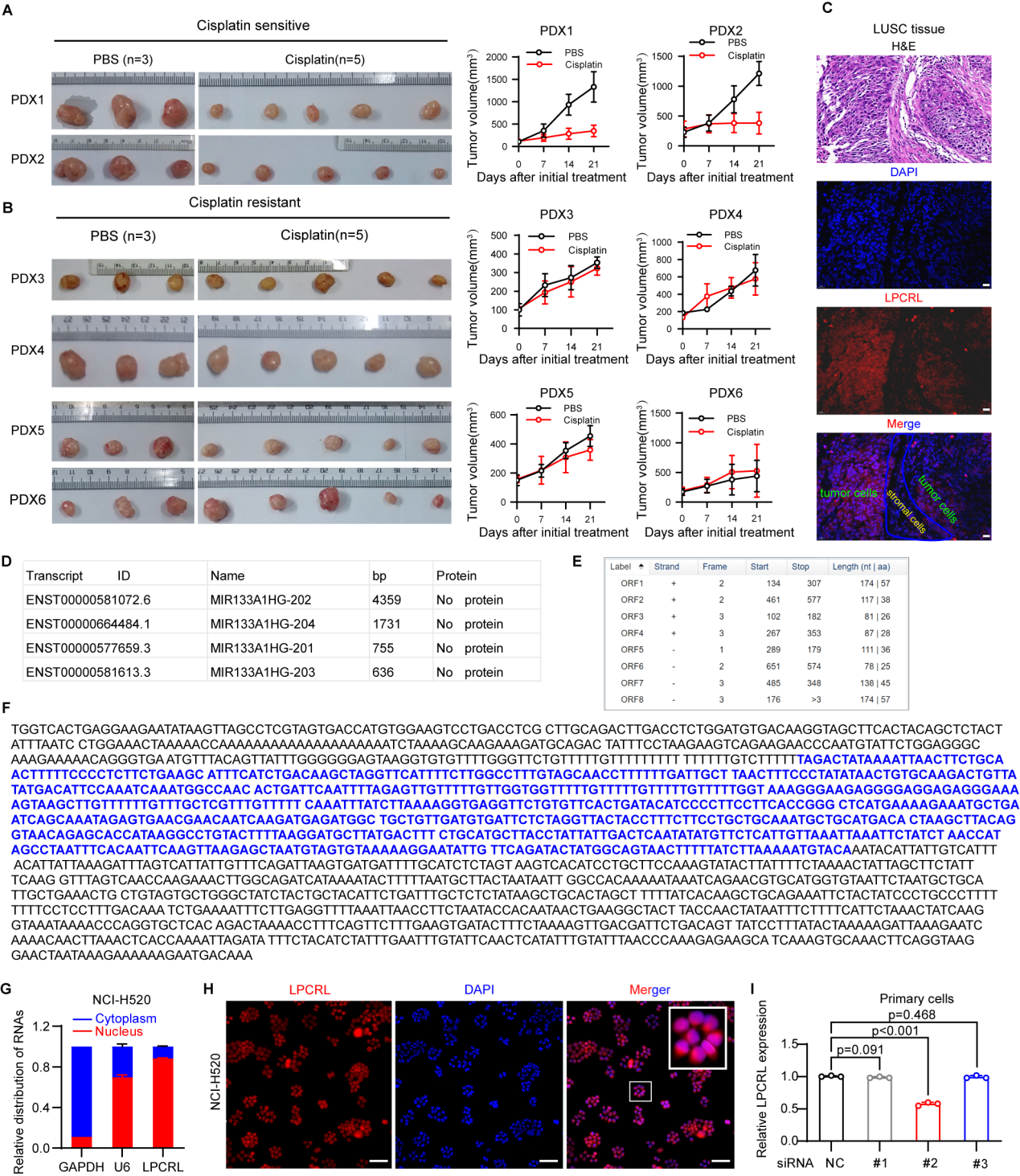
****

### ****Figure S1.  Characterization of PDX models and molecular identification of LPCRL.** (A-B)** In vivo responses of six patient-derived xenograft (PDX) models to cisplatin. (A) Cisplatin-sensitive models (PDX1 and PDX2). (B) Cisplatin-resistant models (PDX3–PDX6). Representative images of excised tumors (left panels) and corresponding tumor growth curves (right panels) from the cisplatin and PBS groups for each model are shown. **(C)** Representative H&E staining (upper panel) and fluorescence in situ hybridization (FISH) images (lower panel) showing LPCRL signals (red) in tumor sections. Nuclei are counterstained with DAPI (blue). Scale bar, 20 μm. **(D)** Four transcript variants of the host gene MIR133A1HG, as displayed in the UCSC Genome Browser. **(E)** Schematic of the full-length MIR133A1HG-204 transcript (1,731 nt), with the identified LPCRL sequence (688 nt) highlighted in blue. **(F)** A screenshot from ORF Finder shows predicted open reading frames (ORFs) within the 688-nucleotide LPCRL sequence. **(G)** Subcellular localization of LPCRL in NCI-H520 cells based on nuclear-cytoplasmic RNA fractionation followed by RT-qPCR. GAPDH (cytoplasmic) and U6 (nuclear) served as controls. **(H)** Representative fluorescence in situ hybridization (FISH) images showing LPCRL signals (red) in NCI-H520. Nuclei are stained with DAPI (blue). Scale bar: 50 μm. **(I)** Validation of LPCRL knockdown efficiency by RT-qPCR in primary cells transfected with negative control siRNA (si-NC) or three independent LPCRL-targeting siRNAs (si-LPCRL #1, #2, #3).

**
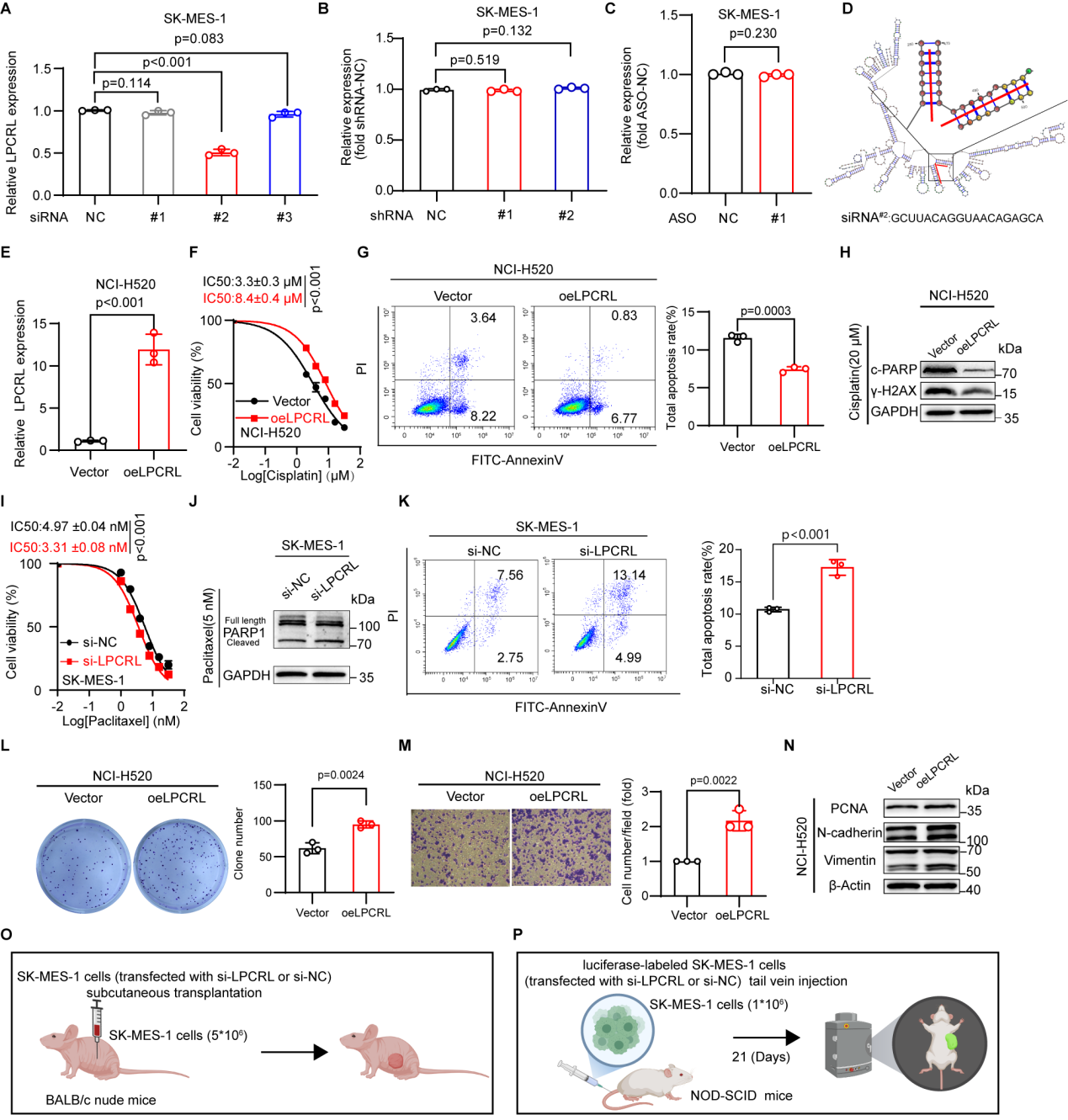
**

****Figure S2.** LPCRL promotes cisplatin resistance, cell proliferation and migration** **in NCI-H520 cells (A-C)** RT-qPCR analysis of LPCRL expression in SK-MES-1 cells transfected with LPCRL-targeting siRNA (A), shRNA (B), and ASO (C). **(D)** Schematic illustrating the target site of siRNA (siRNA^#2^) on LPCRL. **(E)** RT-qPCR analysis of LPCRL expression in NCI-H520 cells stably overexpressing LPCRL (oeLPCRL) or transfected with empty vector (Vector). **(F)** Cell viability was evaluated by MTT assay in NCI-H520 cells stably overexpressing LPCRL (oeLPCRL) or transfected with empty vector (Vector) following 48-hour treatment with various concentrations of cisplatin. **(G)** Representative Annexin V-FITC/PI staining images showing apoptosis in NCI-H520 cells stably overexpressing LPCRL (oeLPCRL) or transfected with empty vector (Vector) following 24-hour treatment with 20 μM cisplatin, along with quantification of apoptotic rates (right panel), are presented. **(H)** Western blot analysis of protein levels of γ-H2AX (a DNA damage marker) and cleaved PARP1 (c-PARP; an apoptosis marker) in NCI-H520 cells stably overexpressing LPCRL (oeLPCRL) or transfected with empty vector (Vector) following 48-hour treatment with 20 μM cisplatin. **(I)** Cell viability was evaluated by the MTT assay in SK-MES-1 cells transfected with si-LPCRL or si-NC, followed by treatment with various concentrations of paclitaxel for 48 hours. **(J)** Western blot analysis showing the protein expression of full-length/cleaved PARP1 (a marker of apoptosis) in SK-MES-1 cells transfected with si-LPCRL or si-NC following 48 h of 5 nM paclitaxel treatment**. (K)** Representative Annexin V-FITC/PI staining images (left panel) showing apoptosis in si-LPCRL- or si-NC-transfected SK-MES-1 cells after 24 hours of treatment with 10 nM paclitaxel and quantification of apoptotic rates (right panel). **(L-M)** Colony formation (L) and Transwell assays (M) assessing the proliferative and migratory capacities of NCI-H520 cells stably overexpressing LPCRL (oeLPCRL) or transfected with empty vector (Vector). Representative images (left panel) and quantification (right panel) are presented. **(N)** Western blot analysis of protein levels of PCNA, N-cadherin, and Vimentin in NCI-H520 cells stably overexpressing LPCRL (oeLPCRL) or transfected with empty vector (Vector). **(O-P)** Schematics illustrating the generation of subcutaneous tumor models (O) and lung metastasis models (P).


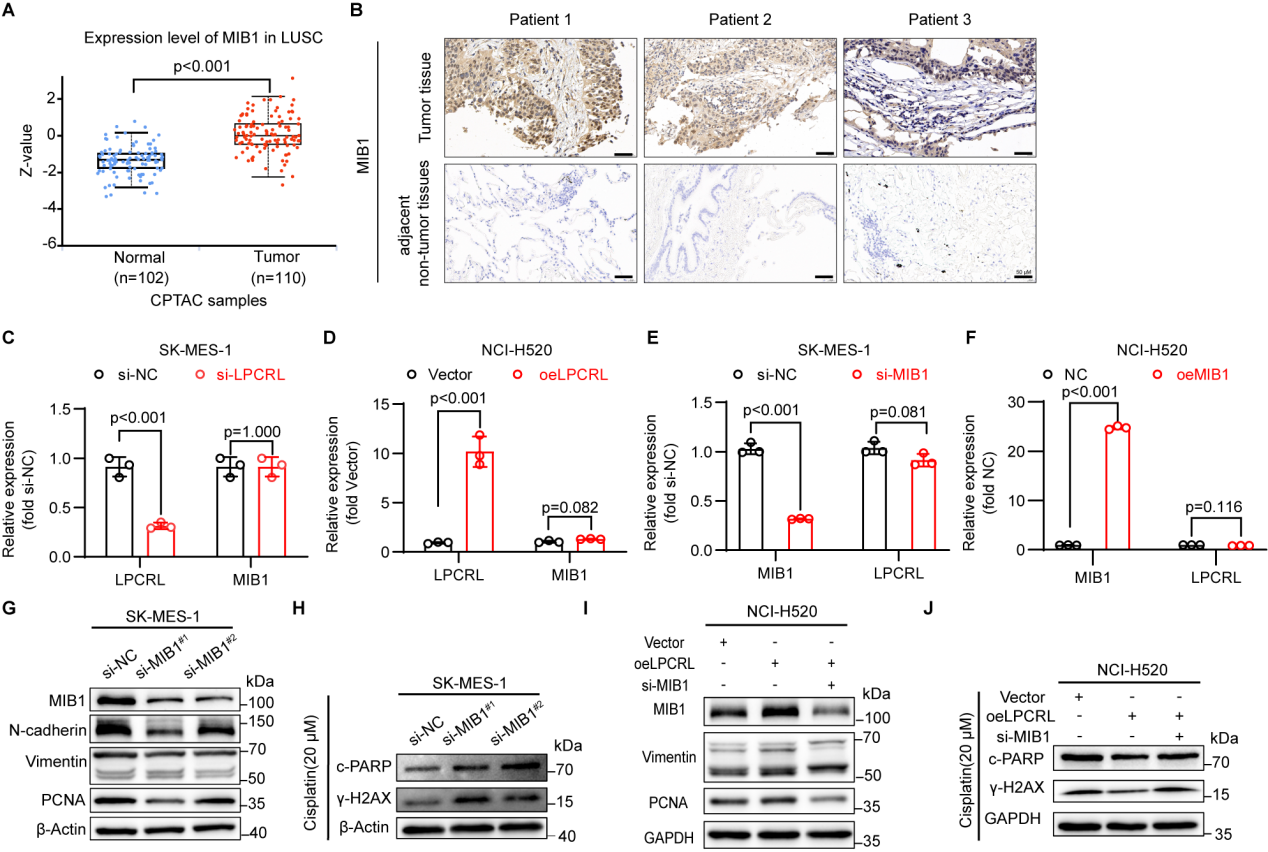


****Figure S3. MIB1 is highly expressed in lung squamous cell carcinoma tissues.** (A)** Analysis of MIB1 protein levels in LUSC and adjacent normal tissues using the CPTAC database. **(B)** Representative immunohistochemical (IHC) staining images of MIB1 in clinical LUSC tissues and matched adjacent normal tissues. Scale bar, 50 μm. **(C-D)** RT-qPCR analysis of MIB1 mRNA levels in SK-MES-1 cells following LPCRL knockdown (C) and in NCI-H520 cells following stable LPCRL overexpression (oeLPCRL) (D). **(E-F)** RT-qPCR analysis of LPCRL expression in SK-MES-1 cells following MIB1 knockdown (E) and in NCI-H520 cells following MIB1 overexpression (F). **(G)** Western blot analysis of MIB1, PCNA, N-cadherin, and vimentin protein levels in SK-MES-1 cells following MIB1 knockdown. **(H)** Western blot analysis of γ-H2AX and cleaved PARP (c-PARP; an apoptosis marker) protein levels in SK-MES-1 cells with or without MIB1 knockdown (siMIB1 or siNC) following 48-hour treatment with cisplatin (20 μM). **(I)** Western blot analysis of vimentin and PCNA protein levels in NCI-H520 cells (oeLPCRL + si-MIB1). **(J)** Western blot analysis of γ-H2AX and cleaved PARP (c-PARP; an apoptosis marker) protein levels in NCI-H520 cells (oeLPCRL + si-MIB1) following 48 hours of treatment with cisplatin (20 μM).


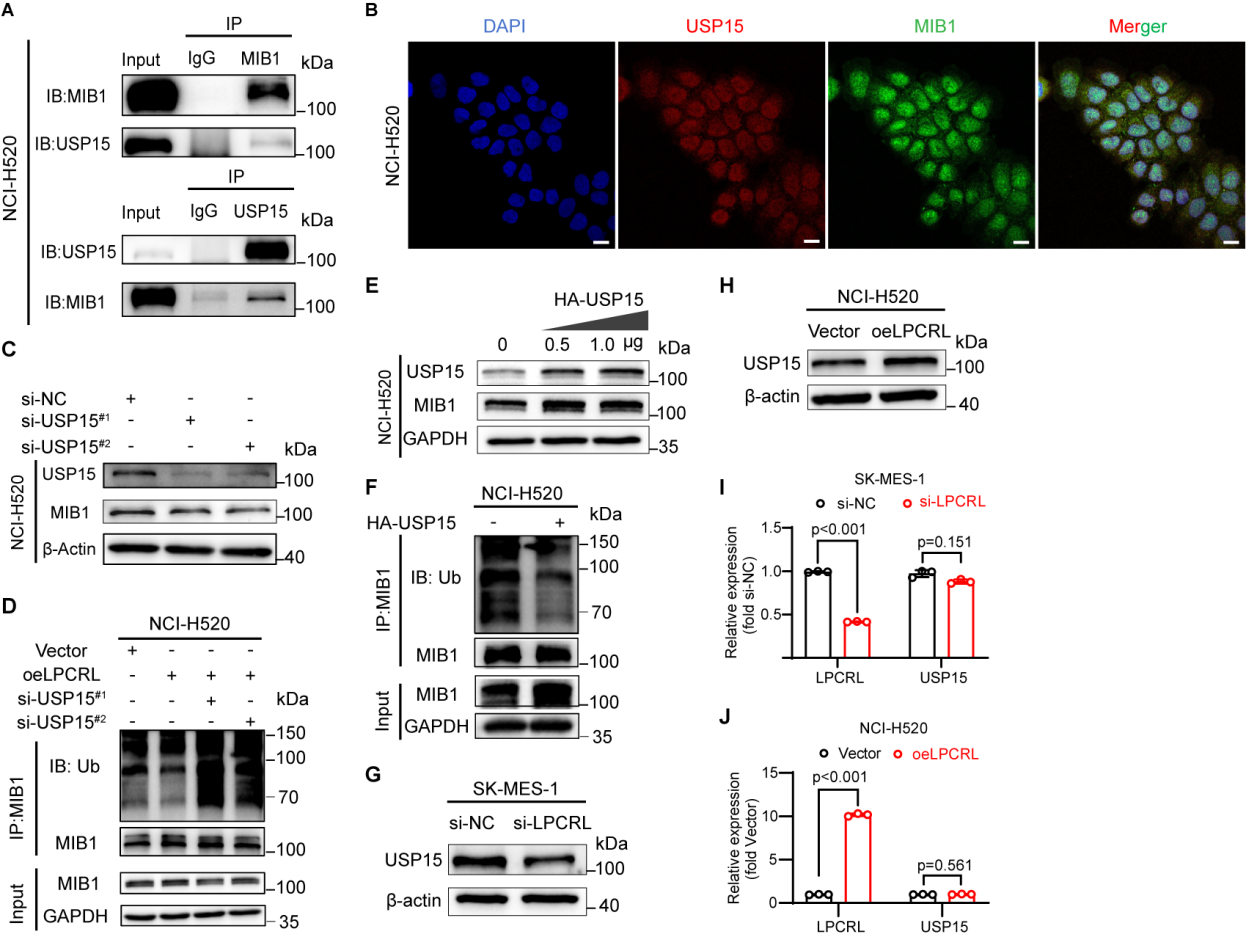


****Figure S4. Endogenous interaction between LPCRL, MIB1 and USP15 in LUSC cells. (A)** Co-IP showing the endogenous interaction between MIB1 and USP15 in NCI-H520 cells. **(B)** Representative immunofluorescence images of endogenous MIB1 (green) and USP15 (red) in NCI-H520 cells; nuclei were stained with DAPI (blue). Scale bar: 10 µm. **(C)** Western blot analysis of MIB1 protein levels in NCI-H520 cells transfected with si-USP15 or si-NC. **(D)** Western blot analysis of MIB1 ubiquitination levels in NCI-H520 cells stably overexpressing LPCRL (oeLPCRL) with USP15 knockdown (si-USP15). **(E-F)** Western blot analysis of MIB1 protein levels (E) and its ubiquitination (F) in NCI-H520 cells transfected with USP15-expressing plasmids. **(G-H)** Western blot analysis of USP15 protein levels in SK-MES-1 cells transfected with si-LPCRL or si-NC (G) and in NCI-H520 cells stably overexpressing LPCRL (oeLPCRL)** or transfected with empty vector (Vector) **(H). **(I-J)** RT-qPCR analysis of USP15 mRNA levels in SK-MES-1 cells transfected with si-LPCRL or si-NC (I) and in NCI-H520 cells stably overexpressing LPCRL (oeLPCRL)** or transfected with empty vector (Vector) **(J).**


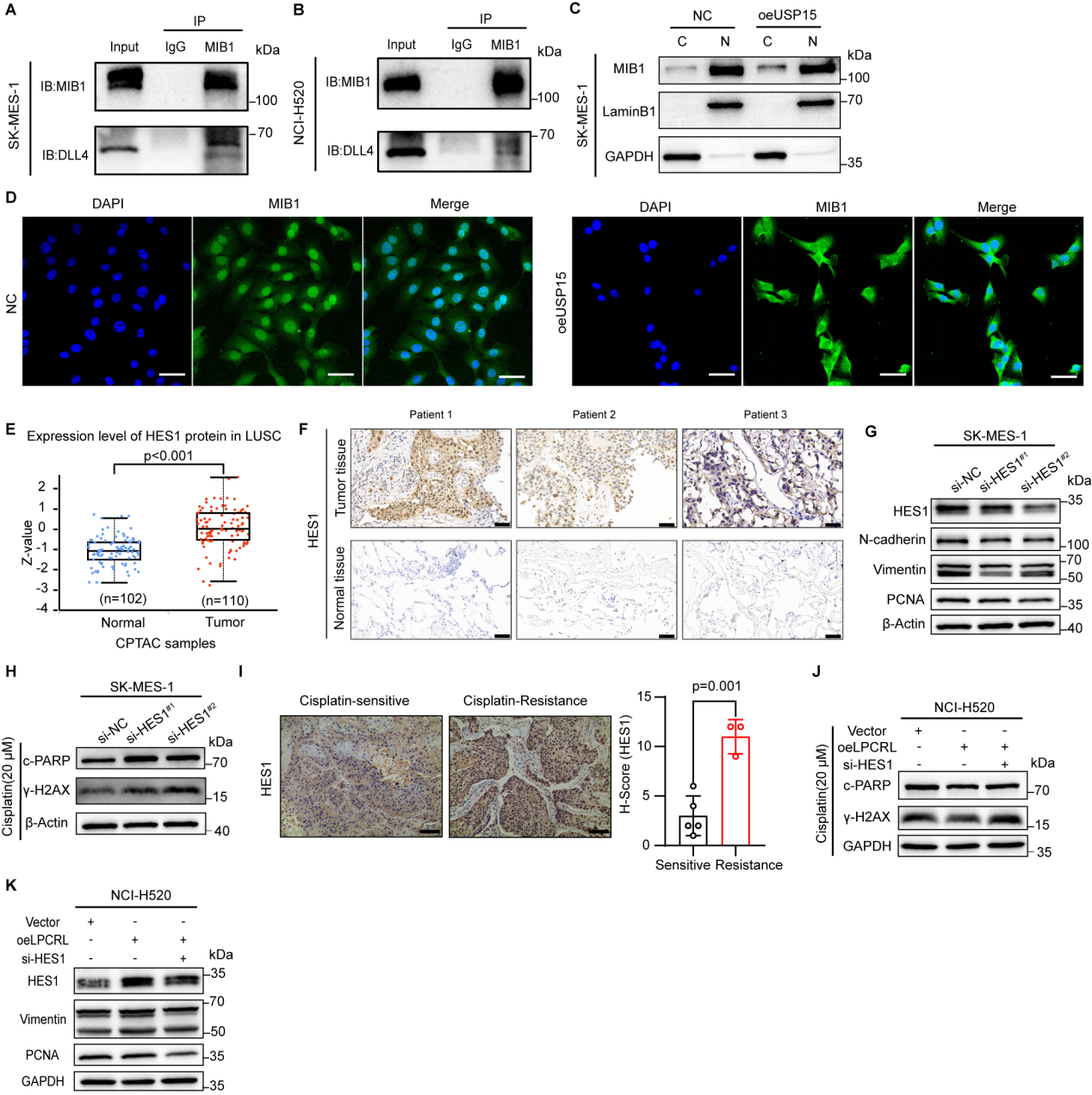


****Figure S5. LPCRL-MIB1-USP15 complex activates Notch signaling pathway.** (A-B)** Co-IP assay showing the interaction between MIB1 and DLL4 in SK-MES-1 (A) and NCI-H520 cells (B). **(C)** Western blot analysis of MIB1 protein levels in subcellular fractions from SK-MES-1 cells with or without transient USP15 overexpression. **(D)** Representative immunofluorescence images showing subcellular localization of MIB1 (green) in SK-MES-1 cells with or without transient USP15 overexpression; nuclei were counterstained with DAPI (blue). Scale bar: 20 µm. **(E)** Analysis of HES1 protein levels in LUSC and adjacent normal tissues using the CPTAC database. **(F)** Representative immunohistochemical (IHC) staining images of HES1 in clinical LUSC tissues and matched adjacent normal tissues. **(G)** Western blot analysis of HES1, PCNA, N-cadherin, and Vimentin protein levels in SK-MES-1 cells following HES1 knockdown (si-HES1). **(H)** Representative IHC staining images of HES1 in cisplatin-resistant and cisplatin-sensitive patient-derived xenograft (PDX) tissues; quantitative analysis of HES1 by H-score is shown (right panel). Scale bar: 20 µm. **(I)** Western blot analysis of γ-H2AX and cleaved PARP (c-PARP; an apoptosis marker) protein levels in SK-MES-1 cells with HES1 knockdown (si-HES1) following 48-hour treatment with cisplatin (20 μM). **(J)** Western blot analysis of γ-H2AX and cleaved PARP (c-PARP; an apoptosis marker) protein levels in NCI-H520 cells stably overexpressing LPCRL (oeLPCRL) with HES1 knockdown (si-HES1) following 48-hour treatment with cisplatin (20 μM). **(K)** Western blot analysis of Vimentin and PCNA protein levels in NCI-H520 cells stably overexpressing LPCRL (oeLPCRL) with HES1 knockdown (si-HES1).


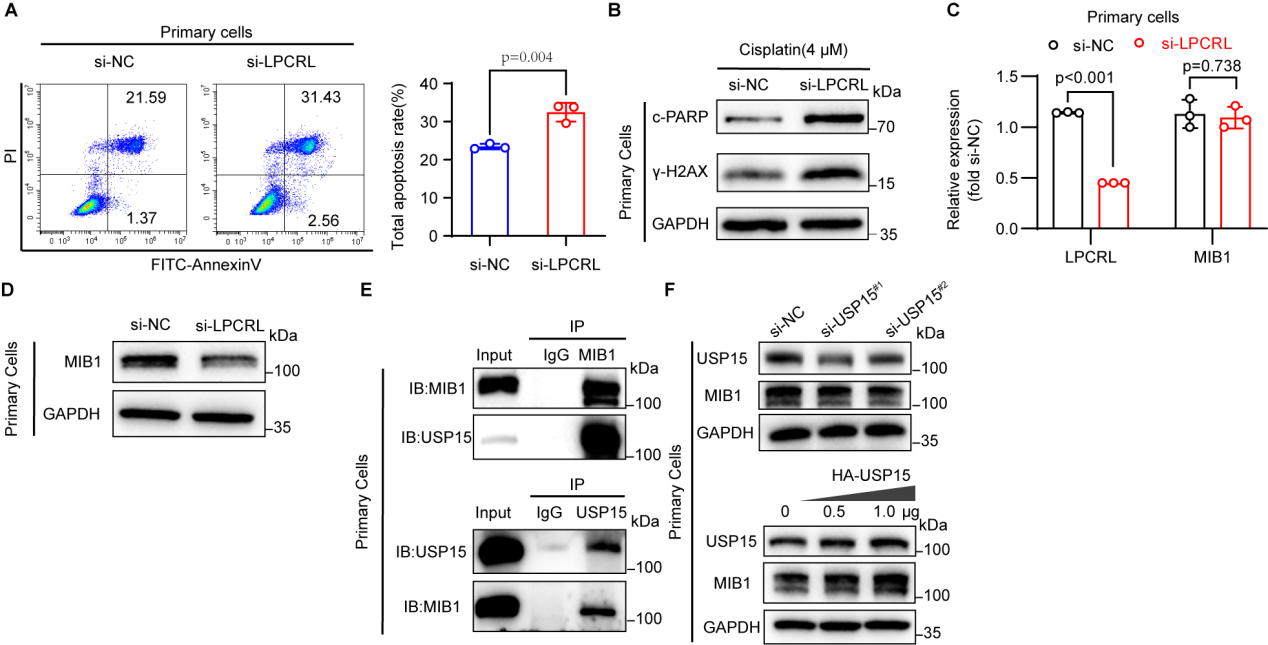


**Figure S6. Therapeutic targeting of LPCRL in primary LUSC cells.** Primary LUSC cells were transfected with si-LPCRL or si-NC, followed by analysis via MTT, colony formation, flow cytometry, RT-qPCR, and western blot assays. **(A)** Apoptosis distribution was evaluated by flow cytometry after 24-hour cisplatin treatment (10 μM) using CytExpert software. Representative Annexin V-FITC/PI staining images and apoptotic rate quantification (right panel) are shown. **(B)** Cleaved PARP (c-PARP) and γ-H2AX protein levels were analyzed by western blot following 48-hour cisplatin treatment (4 μM). **(C–D)** MIB1 mRNA (C) and protein (D) levels were measured by RT-qPCR and western blot, respectively. **(E)** Endogenous interaction between MIB1 and USP15 in primary cells was detected by Co-IP. **(F)** MIB1 protein levels in primary cells following USP15 knockdown (si-USP15 or siNC) or overexpression (USP15-expressing plasmid (HA-USP15) or empty vector) were analyzed by western blot.
